# Current status and progress in research on dressing management for diabetic foot ulcer

**DOI:** 10.3389/fendo.2023.1221705

**Published:** 2023-08-17

**Authors:** Pingnan Jiang, Qianhang Li, Yanhong Luo, Feng Luo, Qingya Che, Zhaoyu Lu, Shuxiang Yang, Yan Yang, Xia Chen, Yulan Cai

**Affiliations:** ^1^ Department of Endocrinology and Metabolism, the Second Affiliated Hospital of Zunyi Medical University, Zunyi, China; ^2^ Department of Endocrinology and Metabolism, Affiliated Hospital of Zunyi Medical University, Zunyi, Guizhou, China; ^3^ Department of Endocrinology, Kweichow Moutai Hospital, Renhuai, Guizhou, China

**Keywords:** diabetic foot ulcer, dressing, biomaterial, wound healing, progress

## Abstract

Diabetic foot ulcer (DFU) is a major complication of diabetes and is associated with a high risk of lower limb amputation and mortality. During their lifetime, 19%–34% of patients with diabetes can develop DFU. It is estimated that 61% of DFU become infected and 15% of those with DFU require amputation. Furthermore, developing a DFU increases the risk of mortality by 50%–68% at 5 years, higher than some cancers. Current standard management of DFU includes surgical debridement, the use of topical dressings and wound decompression, vascular assessment, and glycemic control. Among these methods, local treatment with dressings builds a protective physical barrier, maintains a moist environment, and drains the exudate from DFU wounds. This review summarizes the development, pathophysiology, and healing mechanisms of DFU. The latest research progress and the main application of dressings in laboratory and clinical stage are also summarized. The dressings discussed in this review include traditional dressings (gauze, oil yarn, traditional Chinese medicine, and others), basic dressings (hydrogel, hydrocolloid, sponge, foam, film agents, and others), bacteriostatic dressings, composite dressings (collagen, nanomaterials, chitosan dressings, and others), bioactive dressings (scaffold dressings with stem cells, decellularized wound matrix, autologous platelet enrichment plasma, and others), and dressings that use modern technology (3D bioprinting, photothermal effects, bioelectric dressings, microneedle dressings, smart bandages, orthopedic prosthetics and regenerative medicine). The dressing management challenges and limitations are also summarized. The purpose of this review is to help readers understand the pathogenesis and healing mechanism of DFU, help physicians select dressings correctly, provide an updated overview of the potential of biomaterials and devices and their application in DFU management, and provide ideas for further exploration and development of dressings. Proper use of dressings can promote DFU healing, reduce the cost of treating DFU, and reduce patient pain.

## 1 Introduction

Patients with diabetes are prone to complications of the kidney, retina and nervous system, and approximately 34% of patients have diabetic foot ulcer (DFU). A DFU is defined as a break of the epidermis and at least part of the dermis in a person with diabetes (1). DFU is associated with numerous risk factors and has complex mechanisms and insignificant clinical manifestations. Its pathogenesis is roughly categorized into peripheral neuropathy, Peripheral arterial disease and infection. The pathophysiology of ulcers is also categorized into pre-ulcer, ulcer phase, and ulcer recurrence based on the chronological order of their appearance. DFU is often not detected until it has progressed to an irreversible ulcer. There are about 4 million new DFU patients in China every year, and according to statistics, there is one amputation due to DFU every 30 seconds, accounting for 68% of the non-traumatic amputation population. Moreover, DFU is often accompanied by severe infection, resulting in long-term wound nonhealing, and approximately half of patients with DFU experience lower limb amputation (2). Patients with DFU have a higher risk of death compared to diabetic patients without comorbid DFU (3). The shortened lifespan of DFU patients places a heavy burden on public health and on health care systems. Progress in the development of modern dressings for DFU continues to be driven by the seriousness and urgency of the above situation as well as by extensive clinical and laboratory experience.

The concept of moist wound healing has been accepted by clinical researchers since the 1970s. A humid environment promotes autolytic debridement, stimulates collagen production, promotes the migration of keratinocytes to the wound surface, and supports the function of growth factors in the wound microenvironment, thereby reducing pain, inflammation, necrosis, and scarring. This has led to the rapid development of a variety of wet dressings, including hydrogels, hydrocolloids, films, alginates, and foams (4, 5). Clinical practice has become increasingly reliant on wet dressings, and wet dressings are gradually replacing dry dressings such as gauze and bandages. Second, based on the poor prognosis of DFU after multiple microbial infections, the progress of antibacterial dressings will also be reviewed separately. Moreover, wet dressings are becoming increasingly microscopic and have begun to integrate the modern technology used in drug delivery systems.

**Figure 1:**
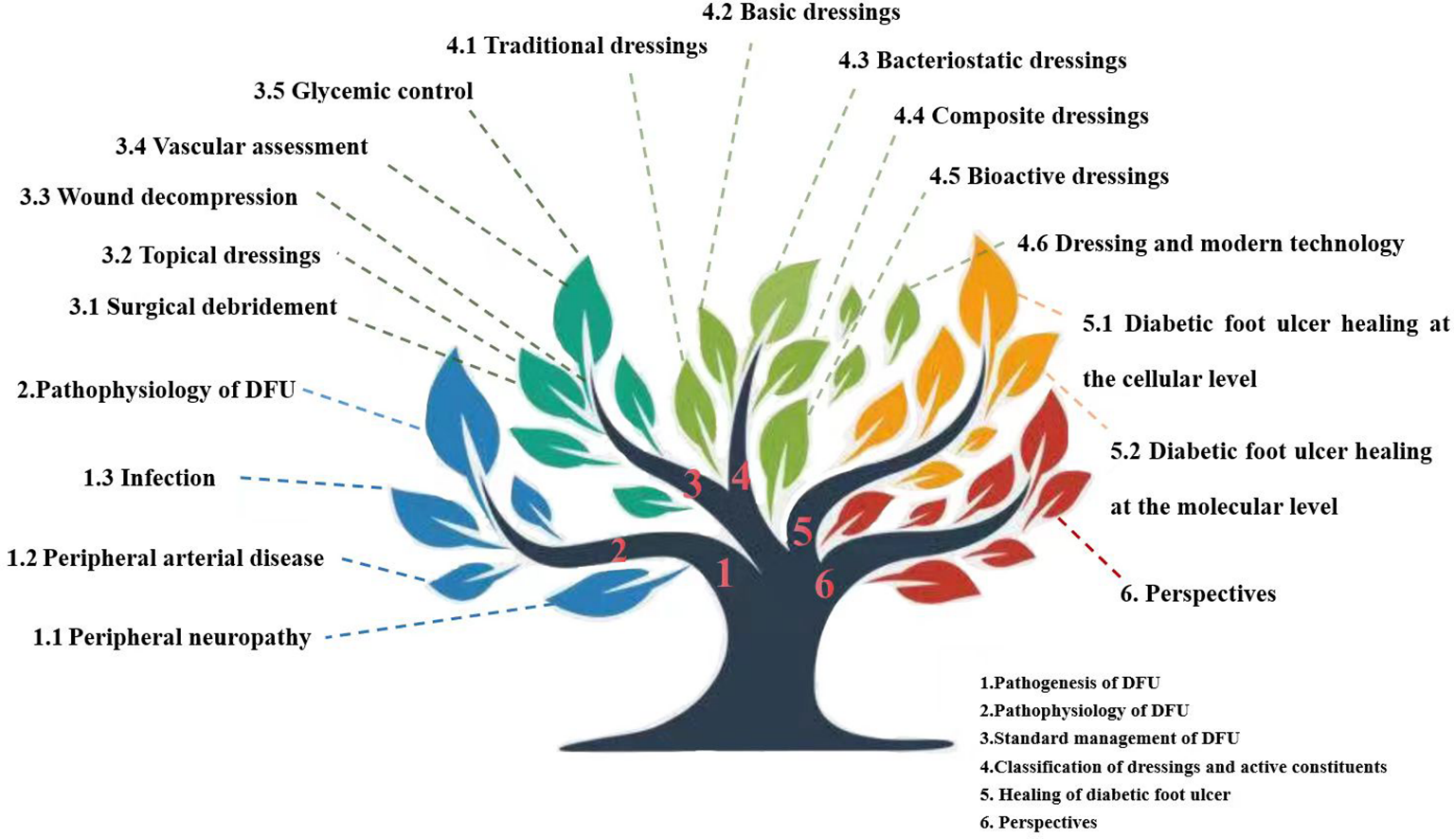
Diagram showing the structure of this review.

Nanodressings, microneedle dressings, bioactive dressings, and dressings produced by 3D printing and photoelectric effects have been developed. Furthermore, modern dressings focus on the monitoring and response of wounds in real time rather than simply on therapy. In fact, prior to the advent of wet dressings, early forms of bioactive dressings such as allografts and xenografts were used. We classify dressings according to their active ingredients (**Figure 1**).

## 2 Pathogenesis of DFU

**Figure 2:**
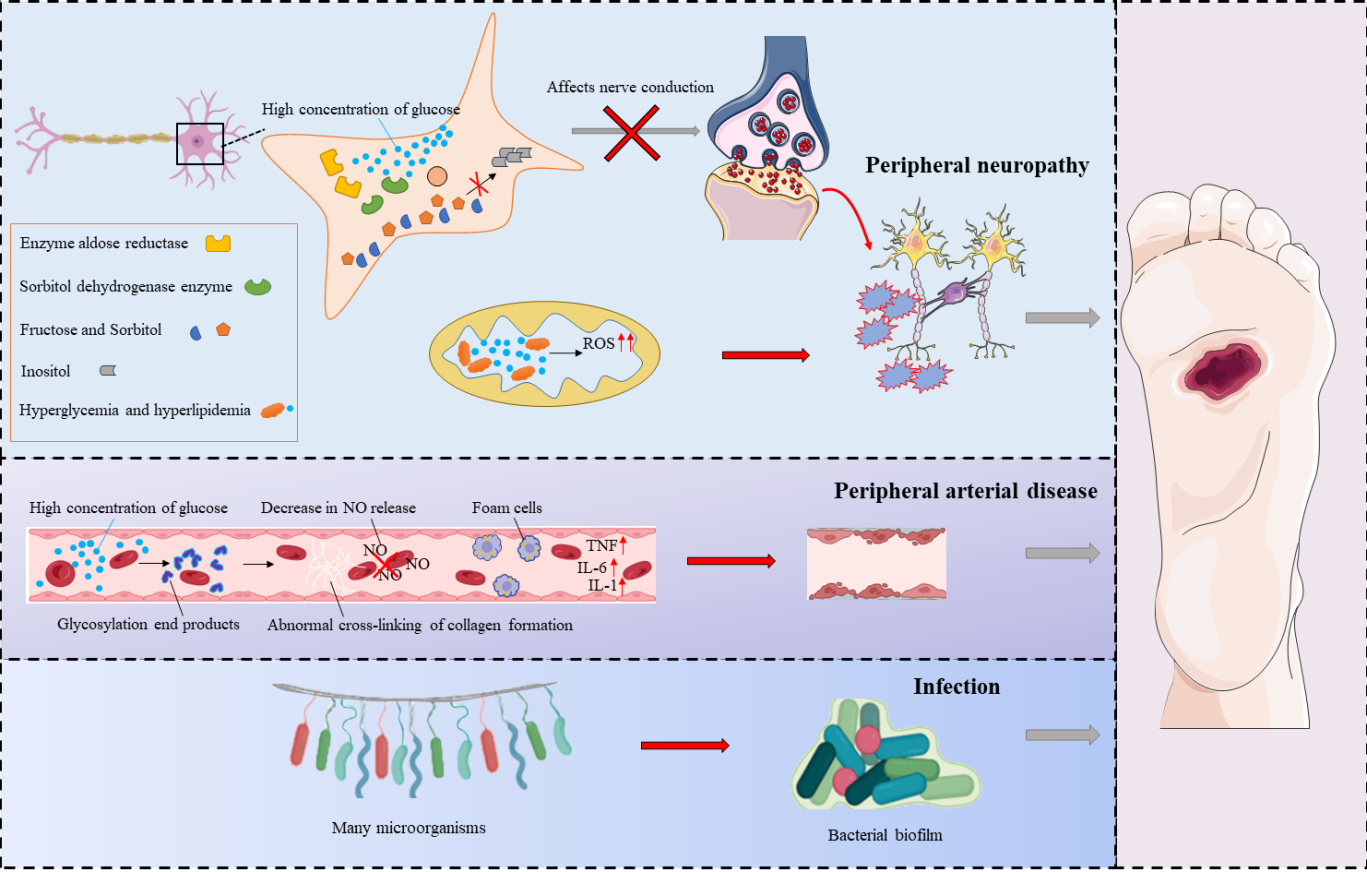
The mechanism of DFU.

There are many risk factors for DFU, and its pathogenesis is very complex. Its pathogenesis can be divided into three categories: peripheral neuropathy, peripheral arterial disease, and infection (6) (**Figure 2**).

### 2.1 Peripheral neuropathy

Diabetic peripheral neuropathy (DPN) is defined as the presence of symptoms and/or signs of peripheral nerve dysfunction in patients with diabetes (7). Neurological disorders associated with diabetes can be classified as sensory, motor or autonomic neuropathy (8). In diseased nerve cells, high concentrations of glucose increase the activities of aldose reductase and sorbitol dehydrogenase, leading to intracellular conversion of glucose to sorbitol and fructose, compounds that affect nerve conduction (9). At the same time, conditions such as hyperglycemia, dyslipidemia and insulin resistance lead to dysregulation of metabolic pathways, and this in turn leads to an imbalance in mitochondrial redox status that results in excessive formation of reactive oxygen species in mitochondria and in the cytoplasm. These conditions lead to loss of axon energy storage and axonal damage, and this aggravates peripheral nerve lesions and causes damage to the nerves in the foot (10). As a result of neuropathy, damage to the lower extremity is often not felt in time, and the lesion remains subject to repeated stress (including prolonged walking or loading). Moreover, neuropathy leads to imbalances in the muscle tissue and to muscle atrophy in the feet of patients with diabetes. Over time, foot deformities such as foot drop, claw foot, and equinus deformity may occur, leading to or aggravating DFU. Autonomic neuropathy affects perspiration and causes abnormal blood circulation in the foot. With the decrease in foot perspiration and the dysfunction of sebaceous glands, the skin becomes dry and keratinized and is more likely to become cracked, leading to infection (11).

### 2.2 Peripheral arterial disease

The high blood glucose concentrations that occur in individuals with diabetes lead to increased oxidative stress responses, increased matrix protein glycosylation, and accumulation of advanced glycation end products (AGEs). With the accumulation of AGEs, protein structure and function change, leading to microvascular and macrovascular disease (12). Studies have confirmed that AGEs cause collagen to form abnormal crosslinks; this leads to vascular stiffness and decreased nitric oxide release from endothelial cells, and the modification of lipoproteins leads to the formation of foam cells. The formation of AGE/AGER (AGE receptor) complexes in endothelial cells induces the production of nuclear factor κB (NF-КB). Thus, the expression of vascular cell adhesion protein 1 (VCAM-1) and proinflammatory cytokines increases. Eventually, endothelial cell function is disrupted, affecting the normal constriction of blood vessels and causing platelet aggregation, endothelial cell proliferation, and atherosclerosis. Vascular lesions affect the supply of blood and oxygen to tissues. Ischemic hypoxia can lead to poor wound healing, worsening of the condition, ulceration, and, in severe cases, avascular necrosis and even amputation (13).

### 2.3 Infection

DFU occurs when normal barrier function is lost and there is an increased risk of foot infection. The bacteria most often associated with DFU include not only gram-positive bacteria such as *S. aureus* (MSSA—methicillin-susceptible *Staphylococcus aureus*, and MRSA—methicillin-resistant *Staphylococcus aureus*), *Streptococcus β-hemolytic* and *C. striatum* but also gram-negative bacteria such as *P. aeruginosa*, *E. coli*, *A. baumannii*, *Proteus* spp., and *Enterobacter* spp. and some anaerobic bacteria that reside more deeply in the wounds, such as *Bacteroides* spp., *Prevotella* spp., *Clostridium* spp., and *Peptostreptococcus* spp (14). Microorganisms gather in specific areas within DFU wounds, where they and grow and multiply, wrapping themselves with extracellular polymers containing polysaccharides and lipids. The polymeric substances (EPS) secreted by the cells embedded in the ulcer include proteins, lipids, nucleic acids, polysaccharides and other components that aggregate with microorganisms to form biofilms. These films give bacteria the ability to adhere to both biotic and abiotic surfaces.

Because biofilms are resistant to antimicrobial agents and to immune and chemical attacks, they delay wound healing and cause chronic inflammation and repeated infections (15). Hyperglycemia reduces leukocyte function, most notably the function of neutrophils, and this is reflected in reduced production of chemokines, increased production of reactive oxygen species, and reduced phagocytosis and migration of cells caused by complement system dysfunction (16). At the same time, keratinocyte migration in DFU wounds is impaired, and this is one of the reasons for slow wound healing (17).

## 3 Pathophysiology of DFU

According to the sequence of appearance of diabetic foot ulcers, their pathophysiology can be roughly divided into pre-ulcer, ulcer phase, and recurrent ulcer phase (18). First, abnormal blood glucose levels in diabetic patients can cause sensory, motor or autonomic neuropathy. The clinical manifestations are loss of sensation, muscle atrophy and deformation, and dry skin. This period is the preliminary stage of foot ulcer development and is also an extremely dangerous period, which can very easily lead to the development of diabetic foot ulcers if not managed properly (e.g., improper patient education). Entering the second stage, ulcers develop due to the loss of self-protection of the patient’s foot and peripheral vascular lesions caused by abnormal blood glucose concentrations, in the presence of a large number of repeated traumas and injuries. The clinical manifestation is the development of foot ulcers, which are very prone to wound infection. Therefore, management during this period is particularly important, and the choice of appropriate adjuvant and surgical approach is a key factor in determining the patient’s prognosis. Finally, as the ulcer heals, the clinical manifestations resolve, but diabetic patients are at an extremely high risk of recurrence. Although surgical or pharmacological treatment can improve the blood supply to the trauma, a complete level of control cannot be achieved for the most fundamental causative factors such as neuropathy, peripheral vascular lesions, and infection. Consequently, diabetic patients in this stage often relapse and develop chronic wounds that do not heal over time. And the correct use of appropriate adjuvants can reduce the recurrence rate and improve the quality of life of patients.

## 4 Standard management of DFU

The ultimate goal of DFU therapy is to bring about wound healing and prevent wound infection, amputation, and decreased quality of life. The standard management of DFU primarily involves surgical debridement, topical dressings, wound decompression, vascular assessment, and glycemic control, among others.

### 4.1 Surgical debridement

Surgical debridement is the surgical removal of nonviable or necrotic tissue from the wound bed and drainage of abscesses, if present. In addition to surgery, there are other methods of debridement such as mechanical debridement, enzymatic debridement and biological debridement, with surgical debridement being the effective and preferred method. Surgical debridement promotes wound healing by accelerating granulation tissue formation and re-epithelialization. Surgical debridement also plays an important role in infection control because necrotic tissue provides a breeding ground for bacterial proliferation. The experts made two recommendations: (a) Patients with diabetes-related foot ulcers should not be sent to the operating room for unnecessary surgical debridement if appropriate sharp debridement can be performed on an outpatient basis, as this is more expensive and resource-intensive, and may actually delay debridement if it can be performed chair-side. (b) Patients with diabetes-related foot ulcers with limb- or life-threatening features (e.g. extensive necrosis, oozing fluid or gas infection) must always be referred urgently for expert surgical opinion to assess the need for surgical intervention to avoid the risk of further deterioration and worsening prognosis (19). Surgical debridement is very commonly used in clinical practice. However, due to the complexity of the pathomechanisms of DFU, monotherapy strategies will result in very low levels of recovery, and combination therapy is more effective. A case report states that a 63-year-old male patient with a DFU was treated and managed with a combination of surgical debridement, maggot therapy, negative pressure wound therapy, and a combination of silver foam dressings. After 3 months and 10 days, the patient’s ulcer had completely healed and was discharged from the hospital in good and stable condition (20).

### 4.2 Topical dressings

Dressings are an integral part of the DFU treatment process. Traditional optimal dressings should have the ability to help relieve symptoms, protect DFU wounds and promote wound healing. A currently accepted wound dressing should also (i) have the ability to promote the tissue reconstruction process by providing thermal insulation, gas exchange, increased drainage, and debris removal; (ii) be biocompatible and not cause allergic or immune reactions; (iii) prevent secondary wound infection; and (iv) be easily removable without causing trauma (21). Because there are different types of wounds and the characteristics of each phase of wound healing differ, there is no single dressing that meets all requirements for use with DFU and can be effectively applied in all cases. There are different types of dressings, and each has its own characteristics. Appropriate application of dressings increases the rate of DFU healing, thereby reducing hospitalization and healing time, and reducing the cost of treating DFU (22). Wound type, patient requirements, and cost should be considered when selecting a dressing. Presently available dressings for DFU can be divided into two categories: traditional dressings and current dressings. **Table 1** presents a comparison of the dressings in these two categories.

**Table 1:** Comparison of traditional and current dressings.

Traditional dressings	Current dressings
Easy access to the raw materials needed for preparation	Excellent insulation ability
Simple preparation process	Promote rapid wound healing
Low cost	Reduce reactive oxygen species in wounds
Fast replacement frequency Prone to tissue adhesion	Slow replacement frequency, long-lasting effect Less prone to tissue adhesion
Extremely likely to carry pathogens Absorption of wound exudate affects the efficacy of the treatment, and exudate leaks rapidly from the dressing Slow deposition of granulation tissue Less effective in relieving pain Local dryness, unable to maintain a humid microenvironment Tends to damage the wound and aggravate pain during replacement Slow onset of action, longer treatment course	Excellent antibacterial effect, can reduce bacterial infections High ability to absorb wound exudate Rapid deposition of granulation tissue Effective in relieving wound pain Excellent moisturizing ability Improves microcirculation and shrinks wounds Rapid onset of action and shortened course of treatment

### 4.3 Wound decompression

The most common pathway to DFUs is the application of excessive mechanical pressure to the non-sensory foot. If the mechanical stress is excessive, it can lead to inflammation, DFU development, and prolonged DFU healing time, which in turn increases the risk of infection, hospitalization, and amputation. Reducing excessive mechanical stress using offloading interventions is a major goal and important prerequisite for promoting healing outcomes and preventing ulceration (23). This process involves reducing the load on the affected areas of the foot by redistributing additional pressure to other areas. Bed rest, wheelchairs, crutches to assist with gait, surgical decompression, total contact casts (TCCs), removable cast walkers (RCWs), and offloading shoes are all common methods. Strong evidence supports the use of non-removable knee-high offloading devices (either TCC or non-removable walker) as the first-choice offloading intervention for healing plantar neuropathic forefoot and midfoot ulcers (24). Despite being the gold standard offloading treatment for plantar DFU, these devices remain underutilized in clinical practice.

### 4.4 Vascular assessment

Up to 50% of patients with diabetes and foot ulcers have coexisting peripheral artery disease (PAD), which leads to a significantly higher risk of adverse limb events and cardiovascular disease (25). Early identification of PAD in patients with diabetic foot ulcers (DFUs) is important because the presence of PAD is associated with an increased risk of nonhealing ulcers, infections, and major limb amputations, as well as cardiovascular complications and increased overall mortality. Assessment of PAD by palpation of the pedal pulse or ankle-brachial index (ABI) is recommended for patients with DFU. An ABI below 0.7 is associated with some degree of arterial insufficiency, and patients with an ABI below 0.4 have severe PAD (26). Patients with noncompressible vessels should undergo additional tests, including toe systolic blood pressure, pulse volume recording, transcutaneous oximetry, or dual-function ultrasound. Abnormalities on any of these secondary tests reliably confirm the diagnosis of PAD.

### 4.5 Glycemic control

The close relationship between blood glucose levels and the progression of diabetic complications has been widely reported in the literature. It has been reported that enhanced glycemic control in patients with diabetes mellitus delays the onset of retinopathy, peripheral neuropathy and nephropathy, which are the major risk factors for DFU, and is therefore positively associated with wound healing (23). It has been shown that proper glycemic control will aid in wound healing during the treatment of diabetic foot ulcers. The study by Xiang et al. suggests that reasonable glycated hemoglobin (HbA1c) targets (ranging from 7.0% to 8.0% during treatment) can promote ulcer healing in patients with DFUs without increasing mortality, especially in patients with better glycemic control on admission (27).

## 5 Classification and active ingredients of dressings

### 5.1 Traditional dressings

Traditional dressings, also known as inert dressings, such as gauze, cotton pads and bandages. It is the most widely used dressing in clinical practice due to its low cost and simple manufacturing process (28). As one of the earliest systems used in the treatment of DFU wounds, traditional dressings provide cushioning that reduces pressure, prevents abrasion, protects the wound, and absorbs small amounts of exudate.

Traditional dressings such as dry gauze, oil gauze, cotton gauze and bandages have played a landmark role in the history of dressing development as effective topical treatments (29–31). These dressings are mainly used to prevent direct contact between the wound and contaminants and to absorb exudate, but they do not directly promote wound healing. In addition, dry dressings tend to adhere to the wound, causing secondary damage to the wound when the dressing is replaced and extending the healing time (30, 32, 33). However, as one of the basic dressings, traditional dressings are still widely used in clinical practice.

Traditional dressings are of great significance, and there would be no advancement in modern dressings without the most basic of dressings. Although traditional dressings do not provide effective healing of the wound. However, it can be used to control diabetic foot infections and to prevent diabetic foot ulcers from continuing to develop. It is the most basic treatment and deserves to be emphasized by primary care doctors, especially for remote and poor areas. So we list three of the most basic and representative dressings, dry gauze, oil gauze and traditional Chinese medicine. And they are described in detail.

#### 5.1.1 Dry gauze

In the treatment of DFU wounds, dry gauze has the effect of covering the wound and isolating it from microorganisms, but it has no antimicrobial activity and does not significantly promote wound healing (34). In addition, dry dressings may cause secondary injury to wounds, and current research in this area tends to focus on the use of multidrug combination therapy to reduce the negative impact of dry dressings on wounds. It is more effective for superficial clean ulcerated wounds.

Studies report that it has been possible to compensate for the shortcomings of dry dressings by functionalizing gauze in ways that give it moisturizing and antibacterial properties. For example, carboxymethylated chitosan that exhibits water solubility, biocompatibility and antimicrobial activity has been synthesized by direct alkylation. Calcium alginate and modified chitosan have also been used as hygroscopic polymerizing agents. The two polymers were applied to the surface of cotton gauze, woven with 40s Ne cotton thread using a mat drying method (35). Studies have also shown that application of a mixture of deacetylated chitosan and petrolatum to sterile gauze followed by drying can be used to prepare chitosan-vaseline gauze (CVG) dressings. CVG dressings are soluble, noncytotoxic and antimicrobial. CVG dressing therapy also increases angiogenesis and the microvascular density of wounds and is therefore a highly promising dressing for wound treatment (36). Thus, the comprehensive function and superior performance of dry gauze play an important role in the treatment of DFU.

#### 5.1.2 Oil yarn

Compared with dry gauze, oil gauze has a unique advantage in that it does not adhere to the wound during the healing process. Dong et al. randomly assigned 22 patients with diabetes to a silver ion dressing group and an oil gauze-silver group. The dressings were changed twice weekly until the DFU healed. The healing outcomes and speed of healing were used as clinical therapeutic indices. The results showed that compared with silver ion dressings, silver-gauze dressings showed better clinical efficacy in the treatment of DFU, especially with respect to ulcer healing speed (37).

Oil yarn has a degree of moisturising power and isolates bacteria and promotes wound healing. However, if it is too thick, it can restrict the exchange function of the skin. This prevents the excretion of metabolic waste, prevents the skin from absorbing oxygen and hinders the skin’s metabolism, which then prevents the wound from healing. Moreover, oil yarn is ineffective in preventing wound infection and has certain limitations related to its ability to manage osmotic fluid leakage.

#### 5.1.3 Traditional Chinese medicine

Traditional Chinese medicine (TCM) foot baths have a long history in the treatment of wounds and are widely used to treat surgical wounds, especially infected wounds. Chinese medicine tonics, which are the essence of TCM, have unique advantages over Western medicine in that they affect multiple targets and have significant clinical efficacy and fewer adverse effects (38). The foot bath decoction (FBD), which is designed for used in a foot bath, is one of the TCM formulas. Its main ingredients are raw rhubarb (Shengdahuang), Coptidis Rhizoma (Huanglian), Fructus Forsythia (Lianqiao), aluminum potassium sulfate (Kufan), and Pseudobulbus Cremastrae Seu Pleiones (Shancigu). All of these TCM have a wide range of pharmacological activities that include anti-inflammatory, antibacterial, and metabolism-promoting activity and improvement of the microcirculation (39). At the same time, certain other TCM adjuvant treatments such as external application, acupuncture, massage, acupoint injection, fumigation and moxibustion also have a certain therapeutic potential for DFU (28). Recent progress in research on TCM-assisted treatment of DFU is summarized in **Table 2**.

**Table 2:** Overview of DFU-assisted therapy with traditional Chinese medicine.

Type	Active ingredient	Mechanism of action	Clinical application
Massage (40)	Administered at specific locations	Changes nerve conduction velocity	Adjunctive therapy for diabetic peripheral neuropathy (DPN) and early DFU.
External application (41, 42)	Compound Phellodendron liquid, ARCC [*Angelica sinensis* (A), Radix Rehmanniae (R), calcined gypsum (C), and calamine (C)]	Upregulates VEGF and PDGF expression in wound tissues to promote angiogenesis, cell proliferation and inhibition of local inflammatory responses	Compound Phellodendron liquid, ARCC
Acupuncture (43)	Acupoint stimulating control	Promotes cell proliferation and angiogenesis, induces extracellular matrix remodeling and reduces inflammation	Encircling needling, Bangci (focal center-side needling), auricular acupuncture, pestle needling therapy, and traditional acupuncture
Moxibustion (44)	Smoke and heat	Promotes the formation of collagen fibers, granulation tissue and capillaries and inhibits inflammation	Moxibustion treatment

In summary, traditional dressings are mainly used to control diabetic foot infections and thus prevent the development of diabetic foot ulcers. Based on previous studies, we conclude that these dressings are suitable for patients with Wagner classification of 2 and 3. The Wagner system assesses ulcer depth and the presence of osteomyelitis or gangrene by using the following grades: grade 0 (pre- or postulcerative lesion), grade 1 (partial/full thickness ulcer), grade 2 (probing to tendon or capsule), grade 3 (deep with osteitis), grade 4 (partial foot gangrene), and grade 5 (whole foot gangrene) (39).

### 5.2 Basic dressings

To overcome some of the shortcomings of traditional dressings, basic dressings have been developed. Basic dressings are made of polymers crosslinked to form a compound with a certain structure. It has better biocompatibility, degradability, and moisture retention and a dressing with strong exudate absorption. As mentioned earlier, dressings with a certain spatial structure facilitate the maintenance of a relatively constant local temperature and humidity in the wound, providing conditions similar to the internal environment of the body (45). Interestingly, basic dressings may avoid re-injury of new granulation tissue due to scar formation and promote cell proliferation, differentiation and epithelial cell migration. In particular, they may play a role in avoiding wound contact with external bacteria and effectively preventing cross-infection (46). Basic dressings have a strong ability to absorb exudate. In addition, they are insulating and impermeable to water and bacteria, making them more comfortable to wear. Moreover, basic dressings do not stick to wounds, making it possible to avoid secondary damage to the wounds during dressing changes and reducing pain. Basic dressings also require fewer changes than conventional dressings (47). Basic dressings include hydrogel dressings, alginate dressings, films (permeable films and membrane dressings), hydrocolloid dressings, sponge foam dressings, capillary‐action dressings, and odor‐absorbing dressings (48). All of these dressings are widely used and effective in DFU treatment. One of the most widely used basic dressings is hydrogel. We describe it in detail and give a brief overview of other basic dressings.

#### 5.2.1 Hydrogel dressings

**Figure 3:**
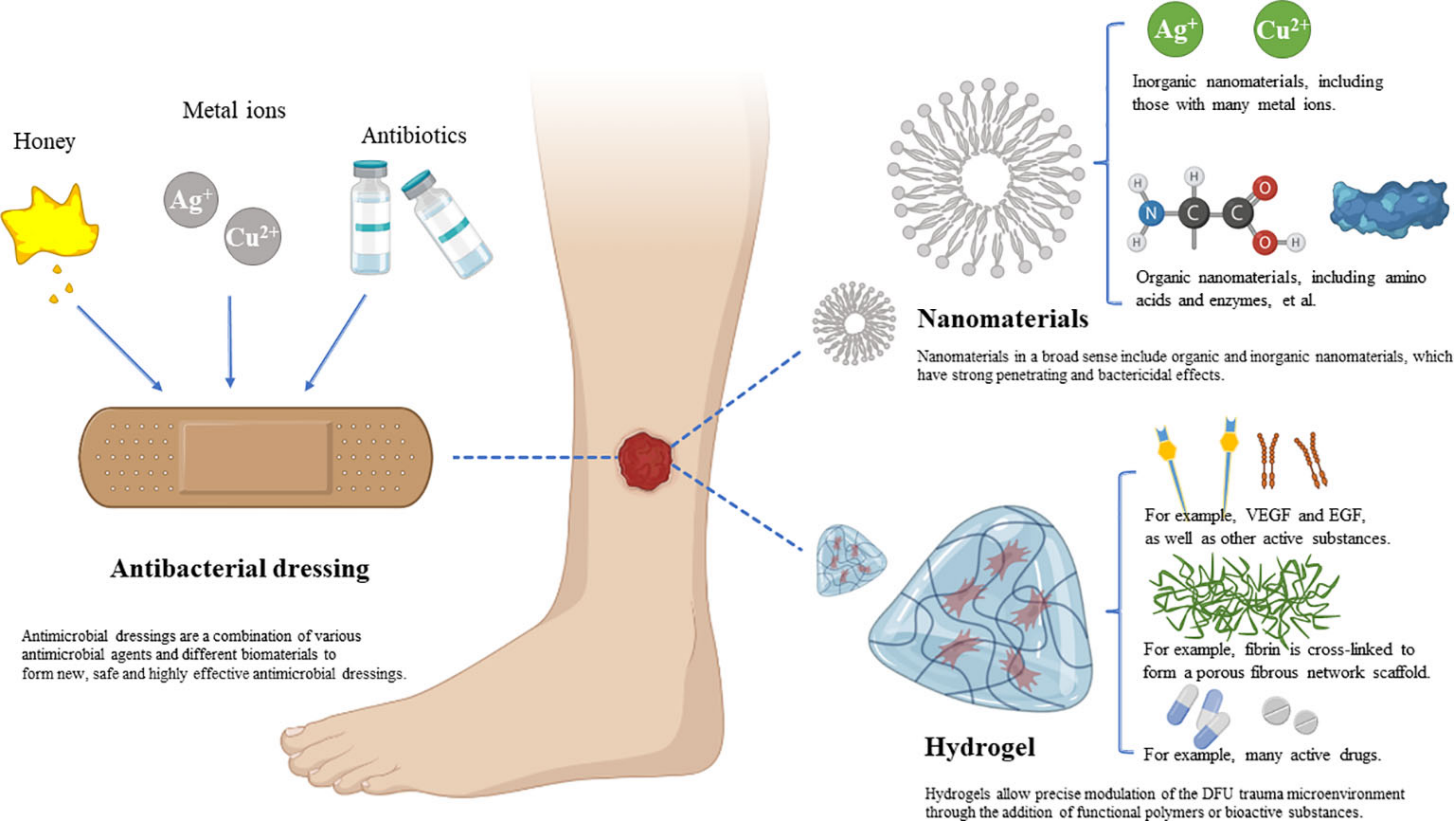
Schematic diagram showing antibacterial dressings, nanodressings and hydrogel dressings.

As a new biomaterial, hydrogels are essentially insoluble hydrophilic polyurethane polymers. They are widely used in the treatment of DFU wounds because of their moisturizing properties, biocompatibility and similarity to living tissue, properties that allow hydrogels to produce the best wound healing effect. The hydrophilicity of a hydrogel, which is a three-dimensional (3D) network structure with high water content, depends on the degree of crosslinking of its polar functional groups. The hydrogel is in direct contact with the wound surface, and its three-dimensional network structure promotes the absorption and retention of water. This long-term moistening of the wound environment helps maintain gas exchange, cell migration and tissue regeneration within the wound and promotes wound healing (49–52). At the same time, hydrogels do not adhere to wounds, are easy to apply and remove without secondary damage and are considered ideal DFU dressings (53–56) (**Figure 3**). Moreover, based on the special structure of hydrogels, precise regulation of the DFU wound microenvironment can be achieved by adding functional polymers or bioactive substances, and these modifications can help accelerate wound healing and promote the healing of difficult-to-heal wounds (57). When used as drug delivery systems, hydrogels can improve the efficiency of drug delivery while minimizing the toxic damage to wounds that is sometimes caused by drugs (58). However, the drug delivery systems that can be created using hydrogels are also somewhat flawed. If only a single extracellular matrix (ECM) component (gelatin, collagen, or hyaluronic acid) is present in the gel, the potential to provide the optimal microenvironment for the wound is limited.Existing hydrogel dressings cannot meet all the requirements for DFU wound treatment; therefore, different drugs must be used at various stages of wound healing (59, 60). The functional hydrogels were designed by simulating the ECM microenvironment. According to the characteristics of functional hydrogels, functional hydrogels can be divided into anti-inflammatory hydrogels, antioxidant hydrogels (AOH), antibacterial hydrogels (ABH), and proangiogenic hydrogels. According to the meta-analysis, early treatment with AOH followed by ABH a week later could be an advanced strategy for future DFU treatment. This information is important for researchers and/or physicians considering the alternative application of hydrogel dressings (61).

It is well known that the inflammatory response is an important obstacle to the healing of DFU wounds. Hydrogels can be classified as those that contain anti-inflammatory agents, those that are based on anti-inflammatory materials and those that contain anti-inflammatory biological components (62, 63). For example, hydrogels containing ibuprofen (IBU), a nonsteroidal anti-inflammatory drug (NSAID) that acts as an anti-inflammatory agent by inhibiting immune cell aggregation and platelet aggregation, have been widely used (64). Research shows that sacran hydrogel membranes can improve skin barrier function, regulate the production of anti-inflammatory cytokines, and achieve anti-inflammatory effects and that they therefore have potential value in promoting wound healing (65–67). Hydrogels that contain biological components, such as fibrin hydrogels, counteract the inflammatory response by forming porous fibrous network scaffolds through fibrin crosslinking; these scaffolds promote infiltration by and aggregation of anti-inflammatory macrophages (68).

The paragraph above discussed the use of anti-inflammatory hydrogel dressings in the treatment of chronic wounds. The following paragraph discusses the application of AOH dressings and proangiogenic hydrogels to chronic wounds. Some researchers have designed functional hydrogels that simulate the ECM microenvironment. As functional hydrogels, antioxidant hydrogels exert antioxidant effects through the presence of curcumin (an antioxidant drug) or other bioactive substances within the hydrogel (69). Vascularized hydrogels that contain bioactive components such as epidermal growth factor or vascular endothelial growth factor can promote the regeneration of blood vessels and subsequently promote the healing of DFU (70). In addition, the three-dimensional network structure of the extracellular matrix simulated by hydrogels can provide shelter for stem cells in the inflammatory microenvironment and maintain the survival and vitality of stem cells in DFU wounds. Compared to treatment with mesenchymal stem cells (MSCs) grown under standard conditions, wounds treated with MSC-seeded hydrogels showed significantly accelerated healing and a return of skin appendages (71). Interestingly, some drugs can also be released by hydrogels as gases. Junpeng Chen et al. developed an all-in-one CO gas therapy-based versatile hydrogel dressing (ICOQF) that produces CO by rapidly removing reactive oxygen from wounds. CO causes oxidative stress, inhibits the synthesis of adenosine triphosphate, exerts antimicrobial effects, inhibits phagocyte proliferation, promotes M1-to-M2 phenotype polarization, and produces anti-inflammatory effects. ICOQF hydrogel is a nonantibiotic antimicrobial dressing that is of great significance considering that global antibiotic resistance is increasing yearly (72). A new study has developed hydrogels based on chitosan (CHT) and the polymer of β cyclodextrin (PCD). Cinnamaldehyde (CN) can be delivered locally at DFU. Antibacterial and antibiofilm activity (*Staphylococcus aureus* and *Pseudomonas aeruginosa*) were evaluated. It was found that the bacteria were reduced by about 99.99% (73).

The hydrogel is hydrophobic, biocompatible, similar to living tissue and does not adhere to the wound. It maintains a moist wound environment and can be used in conjunction with secondary dressings. The precise regulation of the DFU wound microenvironment can be achieved by adding functional polymers or bioactive substances. The addition of antimicrobial components allows it to inhibit bacterial growth and accelerate wound healing. These make hydrogel dressings very versatile and effective for most types of DFU. However, it has some limitations, with its low absorption capacity, poor bacterial barrier and sometimes poor mechanical stability. And it can lead to the accumulation of exudate can lead to wound maceration and bacterial proliferation, requiring the use of different medications at different stages of wound healing, which is more costly. By reviewing the relevant research literature, we learned that hydrogel-based dressings are indicated for patients with Wagner grade 2, 3 or 4 DFU lasting at least 4 weeks. Patients with other high-risk factors were excluded (74).

#### 5.2.2 Other types of basic dressings

Due to their strong ability to resist infection and promote local tissue and cell growth, multifunctional combination dressings are now commonly used clinically (75, 76). Basic dressings other than hydrogels, such as alginate dressings, films (permeable films and membrane dressings), hydrocolloid dressings, sponge foam dressings, capillary-action dressings, and odor-absorbent dressings, are shown in **Table 3**.

**Table 3:** Other types of basic dressings.

Type of dressing	Constituents	Experimental model	Mechanism of action	Clinical effects
Hydrocolloid (77–79)	Semipermeable membranes, foam materials, or nonwoven polyester fibers and hydrophilic biocompatible gel proteins or polysaccharides	Randomized controlled clinical trials involving 535 subjects	Absorbs wound exudate, creates a wet local environment, has a buffer effect	Easy to use, conducive to wound debridement, long maintenance time
Alginate with chlorhexidine hexametaphosphate (CHX-HMP) (80, 81)	CHX-HMP	Wound pathogens were evaluated *in vitro* in terms of total viable count (TVC) and an agar diffusion zone of inhibition (ZOI) model	Absorbs a large amount of wound exudate, prevents leakage, and provides a moist healing environment for the wound surface	At baseline, silver alginate was more effective than CHX-HMP alginate in the TVC test, but CHX-HMP alginate was more effective in the ZOI test
Rubidium-containing calcium alginate hydrogel (82)	Rubidium, calcium alginate hydrogels	*In vitro* experiments on human umbilical vein endothelial cells (HUVECs) and *in vivo* experiments on male SD rats with type II diabetes mellitus were conducted	Kills and inhibits the growth of bacteria, increases the secretion of vascular endothelial growth factor and improves activation of the nuclear factor (erythroid-derived 2)-like 2 (NRF2)/heme-oxygenase-1 (HO-1) signaling pathway	Promotes the migration of fibroblasts and keratinocytes, accelerates neovascularization and epithelial reformation, and improves collagen deposition
Fibracol collagen-alginate wound dressing (83)	Fibracol collagen, calcium alginate	Seventy-five patients with foot ulcers participated in a clinical trial	Absorbs the wound exudate to form a local wet environment and prevents leakage	Collagen-alginate wound dressing is more effective and safer than gauze dressing
Sponge foam (84–86)	Various types of polymers and foam plastics	Clinical trials were conducted on six patients with venous leg ulcers	The sponge foam is pressed by the bandage to achieve even and optimal pressure on the wound bed	Mainly used for mild or high- consumption wounds; can protect and integrate into the skin
Silver-releasing foam dressings (87–89)	Silver	Adult patients diagnosed with type 2 diabetes were selected	Anti-inflammatory and antibacterial	Silver-releasing dressings can significantly reduce the ulcer area in patients with lower limb ulcers and improve the cure rate
Films (48, 90, 91)	Film inclusions (commonly used preservatives such as silver-based compounds, gentamicin sulfate, and other compounds)	Preliminary tests were performed using microspheres with a diameter of 0.71 microns	The inclusion kills bacteria and prevents systemic infection	Single films are only suitable for wounds with a small amount of exudate; the clinical efficacy of combined films is better

### 5.3 Bacteriostatic dressings

For the DFU, an infection would be a catastrophe. Eighty percent of DFU patients have a poor prognosis due to concurrent infection (18). Furthermore, microorganisms infecting DFU wounds are becoming increasingly complex and often resistant to drugs, such as MRSA, which poses a huge challenge to the clinical treatment of DFU. Biofilm formation in a variety of microbial infections protects bacteria from antimicrobial agents and immune responses and is a cause of wound healing failure. It can lead to wound enlargement requiring surgical intervention or even life-threatening. Therefore, there is an urgent need for dressings with anti-infective properties to address this dilemma (92).

To promote wound healing, several drugs (or bioactive agents) are added to the matrix of the dressing preparation, most commonly antimicrobials (93). This has driven the research and application of Bacteriostatic dressings. Combining different antibacterial agents and biological materials to make new antibacterial dressings is currently an active area of research in modern skin tissue engineering. Honey, antibiotics, metals, and metal oxides are the most common pharmaceutical ingredients with antibacterial properties. Biomaterials come in many forms and structures, including thin films, hydrogels, sponges, nanofibers, and other types of structures (94).

In a meta-analysis of 767 patients, patients treated with honey dressings were better than the control group in terms of complete healing rate (RR=1.32, 95% CI: 1.10-1.57, P=0.003), bacterial complete clearance (RR=2.56, 95% CI: 1.33-4.92, P=0.005), mean healing time (SMD=-1.12, 95%CI: -2.06~-0.19, P=0.02). No serious adverse effects were observed (95). Clinical trials have shown that honey contains active enzymes such as glucose oxidase, which produces hydrogen peroxide and inhibits microbial growth (96). Compared to conventional dressing techniques such as iodine voltammetry, honey dressing treatment can significantly better than the control group in terms of pain score, wound pH reduction, antibacterial effect and other aspects (P<0.05), and does not cause blood glucose fluctuations. Clinical confirmation: In the control group, 50 patients with DFU were treated with routine dressing changes. In the treatment group, 50 patients added topical application of honey to this basis. On the 20th day of dressing change, 25 cases of infection occurred in the control group and 18 cases in the treatment group (P<0.05) (97). Therefore, honey dressings can be used clinically as effective and safe antibacterial dressings. The use of a combination of debridement and silver ion hydrogel dressings is another representative anti-infective therapy. Clinically, both nonmechanical (autolysis, enzymatic) and mechanical methods (sharps surgery, wet-to-dry debridement, water-based hyperbaric lavage, ultrasound, negative pressure wound therapy (NPWT), and biosurgery/maggot debridement therapy) are used to debride wounds (98). In NPWT, negative pressure is applied to the wound tissue; this reduces the area of wound exposure and accelerates wound healing by promoting adhesion to the surrounding tissue. The filler used with NPWT is also important; silver ion hydrogel dressings have significantly higher antimicrobial activity than gauze and foam dressings (99). The antimicrobial mechanism of silver ion dressings may be related to their degradation of bacterial cell walls and the promotion of bacterial content outflow. Silver ions also affect the metabolic activity of bacteria by altering the structure of their cell membranes, leading to the death of bacteria that are in an active but nonculturable state (100). Despite this, the use of silver ion dressings for long periods often results in local irritation and decreased compliance among patients. It is therefore necessary to optimize silver nanoparticles (SNPs) for use in wound dressings. The researchers found that sericin- and chitosan-capped silver nanoparticle (S/C-SNP)-loaded hydrogel were more acceptable to patients, and the antimicrobial activity and wound closure exhibited by S/C-SRP were confirmed by histopathological results (101).

The development of antimicrobial dressings based on active polymeric biomaterials has produced unexpected effects. Injectable adhesion-thermosensitive polysaccharide-based dressings (FEPs) deliver exosomes from adipose stromal cells and thereby promote the repair of DFU wounds. The antimicrobial activity of FEP dressings is one of their primary functional properties, especially in cases in which drug-resistant bacteria are present in wounds (102). In addition, a copper (Cu)-containing bioactive glass nanocoating with uniform nanostructure that continuously releases copper ions was prepared on a natural eggshell membrane using pulsed laser deposition (PLD) technology. Copper ions significantly inhibit the survival of bacteria, especially methicillin-resistant *Staphylococcus* and *E. coli*. The presence of copper ions effectively slows the process of bacterial infection (103). A dressing that can be used to rapidly sterilize wounds has also been described in the literature. It contains Ag/Ag@AgCl/ZnO heterogeneous nanostructures embedded in a hydrogel. Exposure of this hydrogel system to simulated visible light kills 95.95% of *E. coli* and 98.49% of *S. aureus* within 20 minutes. In this system, the production of reactive oxygen species is enhanced by exposure to visible light, allowing the Ag/Ag@AgCl nanostructure to enhance the photocatalytic and antibacterial activity of ZnO. The slow release of Ag+ and Zn^2+^ stimulates the immune system, resulting in the production of large numbers of white blood cells and neutrophils. It also produces synergistic antibacterial effects and accelerates wound healing (104). Cross-linked double-network hydrogel biodressings consisting of polyethylene glycol diacrylate (PEGDA) and sodium alginate (ALG) have potent antimicrobial activity and promote healing without any biological agents or drugs. In this innovative dressing design, biomaterials rather than biologics provide antimicrobial activity (105). In summary, the development of antibacterial dressings is aimed at designing and producing safer and more efficient antibacterials.

Typical antibacterial dressings are mainly honey dressings and silver ionomer dressings. The weak acidity of honey inhibits the growth of pathogenic bacteria, thus acting as a cleansing and anti-infective agent, and it also has a strong ability to promote ulcer healing (22). In recent years the use of honey dressings has become more widespread and has proven to be effective. There are many different types of honey and its complex composition needs to be further explored in the future to better guide its clinical application. Silver ion dressing improves wound hygiene and has antibacterial activity. It may cause silver staining on wounds, and silver allergy in some patients limits its use.

### 5.4 Composite dressings

Composite dressing refers to the improvement on the basic dressing by adding polysaccharides, proteins, polymers and other bioactive substances to make the dressing function more perfect. Crosslinking polysaccharides and proteins on top of the base dressing (hydrogel, alginate, film) can form a porous structure. It has many advantages, such as allowing oxygen, drugs, nutrients and metabolic wastes to move in and out of the cell (106). It provides better quality conditions for the healing of DFU. We list the three most commonly used materials for composite dressings and describe them in detail.

#### 5.4.1 Collagen dressings

Normal human skin contains a large amount of collagen, which gives it a tight, intact structure. However, the skin tissue of diabetic individuals contains elevated levels of human matrix metalloproteinases (MMPs) and lysine oxidase (LOX). And the collagen it contains is sparse, disorganized, and prone to breakage. Consequently, the dermal collagen structure is compromised, and the skin appears rough. This abnormal collagen microenvironment may be a risk factor for DFU (107). Therefore, based on the pathological alterations, the development of direct collagen dressings or dressings that promote normal collagen synthesis has great prospective clinical value. In the study, a multifunctional nano and collagen-based materials was designed and applied to animal models of diabetes. When applied to wounds, the antimicrobial nanoparticles first form a layer that prevents bacterial proliferation and eliminates biofilms. After it has been applied, the thermosensitive collagen matrix is plasticized so that it conforms to the wound shape and adheres closely to the wound surface (108). The tensile strength, porosity, and biocompatibility of collagen and its ability to support cell proliferation can be increased using electrochemical deposition methods. Exposure of wounds to thermosensitive collagen increases granulation tissue, epidermal thickness, and reconstruction of tissue. All of these effectively promote wound repair, regardless of whether it binds to adipose-derived mesenchymal stem cells (109). In addition, a porous dressing is made from novel collagen (COL-SPG). In that study, the *in vivo* evaluation of the COL-SPG 3D sponge exhibited with enhanced collagen synthesis and aids in faster reepithelialization (110). In a new study, a bionic, double-layer antibacterial collagen scaffold is reported. It consists of an epidermal anti-bacterial collagen used to prevent wound infections combined with a dermal collagen-glycosaminoglycan scaffold. The dressing exhibits a structure similar to that of natural skin, successfully inhibiting bacterial growth and promoting angiogenesis. This dressing is an excellent candidate for enhancing diabetic wound healing (111).

Collagen is a biocompatible structural protein that is biodegradable and biomimetic, making it an ideal source of biomaterials for tissue engineering and regenerative medicine. Collagen dressings significantly improve wound closure, positively affect unhealed DFU, highly promote angiogenesis and rapid re-epithelialisation (112). There is insufficient evidence to demonstrate the superiority of specific collagen biological sources or combinations. Wound dressings containing collagen appear to have some benefit in the treatment of diabetic foot ulcers and should be carefully considered by the clinician managing the wound.

#### 5.4.2 Chitosan dressings

Chitosan (CS) has received a lot of attention in the field of medical research because of its antibacterial activity, antioxidant activity, high safety, biodegradability, and biocompatibility. CS exists in many forms, such as gels, thin films, and nanoparticles (113, 114). After modification or coupling to other substances, chitosan becomes a wound dressing and a drug delivery system when loaded with active substances (115, 116). The value of chitosan in the treatment of DFU is closely related to its anti-infective and antioxidative properties. For example, hydrogels prepared from chitosan and agarose have pore sizes (90-400 μm) that are compatible with cell internalization and proliferation. Hydrogels containing more than 188 μg/mL chitosan exhibit strong antibacterial properties (50). The antibacterial activities of two types of antimicrobial composite films (CH_2_CuO-CH and CH_2_Cu-CH) made of nanocopper oxide or encapsulated in nanocopper and covered with chitosan (CH) were compared. The results showed that both inhibits the growth of *Escherichia coli* and *Bacillus.* The CH2CuO-CH suppression circle values were 1.0 cm and 0.75 cm, respectively. The suppression circle values of CH2Cu-CH were 0.6 cm and 0.5 cm, respectively. Thus, the nanocomposite CH_2_CuO-CH film shows stronger antimicrobial activity and can be used in antimicrobial applications (117). However, the biological effectiveness of chitosan requires its solubility in water or other solutions, and this limits its widespread use. Ways in which chitosan can be modified to avoid these limiting conditions and enhance its original activity is a focal area of current research. For example, a new family of cationic hydrogels based on arginine-based poly (ester urea urethane) (Arg-PEUU) and glycidyl methacrylate-modified chitosan (CS-GMA) is currently being developed. This modified chitosan dressing accelerates the healing of infected wounds by activating RAW 264.7 macrophages and causing them to increase their release of NO and TNF-α (118). A novel antibacterial hydrogel dressing made of poly(aminoethyl)-modified chitosan (PAEMCS) has also been reported. In antibacterial experiments on *Escherichia coli*, *Staphylococcus aureus*, *Pseudomonas aeruginosa* and *Salmonella*, PAEMCS had higher antibacterial activity than CS at the same concentrations. Experiments have shown that the increase in the number of amino groups increases the antibacterial activity of CS (119). An injectable chitosan-based POSS-PEG hybrid hydrogel has been reported. It contains polyhedral oligosilsesquioxane (POSS), a nanoparticle with excellent stability and biocompatibility. In addition, the effect of hydrogel as a wound repair material in diabetic mice was systematically and comprehensively evaluated by histomorphological analysis using a full-thickness diabetic wound model. The results showed that the hydrogel-treated wound showed faster epithelial tissue regeneration, fewer inflammatory cells, more collagen deposition and higher VEGF expression levels (120). In one study, a novel supramolecular photothermal nanoparticles (MCC/CS NPs) were reported. It consists of mono-carboxyl corrole (MCC) and CS. MCC molecules have good photothermal properties and achieve a photothermal conversion efficiency of 66.4%. Under near-infrared laser irradiation, diabetic wound models of bacterial infection confirmed that MCC/CS NPs can effectively kill drug-resistant bacteria, accelerate wound healing and angiogenesis, and exhibit good biocompatibility (121). Chitosan dressings play an important role in the antimicrobial treatment of DFU.

#### 5.4.3 Nanodressings

Nanomaterials are materials at least one dimension of which (in three-dimensional space) is between 1 and 100 nm in size; this is approximately equivalent to the scale of 10~1000 atoms closely aligned together. Nanoparticles have the property of penetrating the barrier with a small particle size and a high specific surface area. Nanoparticles can interact with biological constituents and infiltrate wound sites. Nanomaterials possess the ability to effectively transport and deliver various pharmacological agents, such as nucleic acids, growth factors, antioxidants, and antibiotics, to specific tissues (122). Specific nanodrug delivery systems can enter the cytoplasmic space or activate specific transport mechanisms, improving drug retention. The incorporation of bioactive molecules prevents drug degradation and enhances therapeutic effects. By using biocompatible and biodegradable nanomaterials, drug delivery systems can be designed to enhance wound healing and provide sustained drug release. Furthermore, nanomaterials can be tailored to meet specific requirements for wound healing, such as enhanced cellular and tissue penetration, antibacterial properties, and controlled mechanical properties. In addition, appropriate antimicrobial action can be achieved by controlling the size and shape of nanopreparations. In wound healing, nanomaterials have shown the potential to promote cell proliferation, migration, angiogenesis, and extracellular matrix remodeling and prevent infections (123). Therefore, nanoparticles are more suitable for many purposes than macroscopic materials.

Nano silver, nano copper, nano copper oxide, nano zinc oxide and nano gold have been widely used in research (124). With the advancement of nanotechnology, it is possible to produce nanoscale sterling silver particles. Silver nanoparticles (AgNPs) is non-toxic to eukaryotic cells but highly toxic to prokaryotic cells. This allows nanosilver to show powerful antibacterial activity. In addition, the antibacterial activity of copper nanoparticles is similar to that of silver nanoparticles. The antibacterial activity of ZnNPs is generally lower than that of AgNPs and Copper NPs. AuNPs have been found to be effective against gram-negative bacteria but less effective against gram-positive bacteria. In a groundbreaking study, the AgNPs were incorporated into carrageenan to develop nanosilver acticoat. *In vivo*, *in vitro* and in silico three-mode studies were carried out. *In vivo* studies showed that dressing with Carrageenan silver nanoparticles (CAgNPs) acticoat promoted wound healing and had good reepithelialization and dense collagen deposition capabilities. *In vitro* experiments were tested against *Escherichia coli* and *Staphylococcus aureus*. Computer analysis provides information about the drug similarity of the dressing and predictions related to human health hazards. The application potential of this dressing in DFU was emphasized (125). Compared with ordinary silver dressing, nano-silver dressing has a larger contact surface and stronger bactericidal effect. In a clinical observation of 160 patients, the patients were randomly divided into groups that received treatment with either epidermal growth factor, a nanosilver dressing, a nanosilver dressing combined with epidermal growth factor, or saline alone, and the time required for wound repair to each healing stage was recorded. The results showed that the wound repair time of the combined nanosilver and epidermal growth factor group was shorter than the repair times of the epidermal growth factor group and the control group, and the differences were statistically significant (126).

Another category of nanomaterials is represented by organic nanomaterials such as self-assembled peptide (SAP) hydrogels made from natural amino acids. SAP hydrogels can be used to create extracellular matrix (ECM)-like nanostructures that mimic the human cellular microenvironment and improve the local lesion state of DFU (127). In the section in which we reviewed collagen dressings, we stated that elevated levels of MMPs in diabetes lead to abnormal collagen deposition. To address this problem, a 3D polycaprolactone (PCL)/collagen (PC) nanofiber dressing (3D-PC) was created that contained the MMP inhibitor doxycycline hydrochloride (DCH) and the antibacterial agent cefadroxiride (CEX). MMPs inhibitors can limit the overexpression of MMPs in DFU wounds to avoid delayed wound healing (128). Multiplex nanoenzymes are another important organic nanomaterial. However, research has been slow due to the incompatible reaction microenvironments of these nanoenzymes and the unsuitability of conventional assembly strategies. Notably, a recent study reported that a fiber-based compartmentalization strategy could be used to provide the preferred microenvironment for each nanozyme. The development of this integrated platform promotes the use of multiplexed nanozymes in DFU therapy (129). Furthermore, a bilayer nanofiber scaffold has been developed (130). The first layer of the multifunctional bilayer nanofiber scaffold (DLS) consists of mupirocin and lidocaine hydrochloride uniformly doped into PCL; the function of this layer is to provide an initial “burst” release of lidocaine hydrochloride followed by slow release of mupirocin. The second layer consists of chitosan. DLS nanofibers are thermally stable, have high antibacterial activity and are nontoxic to fibroblasts (131). In addition to chemicals, herbal extracts have shown unique advantages for use in nanodressings. A study reported the incorporation of *Calendula officinalis* extracts into an electrospun fiber scaffold. The electrospun fiber scaffold consisted of poly(ϵ-caprolactone) (PCL), maize alcoholic protein (Zein), and gum arabic (GA). It exhibits desirable mechanical properties and degradability suitable for skin tissue engineering (132).

Clinical response to wound infections is still dominated by antibiotic therapy. Antibiotic treatment increases microbial resistance over time and often leads to a poor prognosis. It is worth mentioning that nanofiber dressings that do not use antibiotic therapy as a means of treatment are gradually gaining attention. For example, electrospun hyaluronic acid/polyvinyl alcohol/polyethylene oxide blends encapsulated with new ZnO NPs/cinnamon essential oil (CEO) have demonstrated advantages such as good antimicrobial effects, promotion of rapid healing of traumatic injuries, and high safety (133). The remaining inorganic and organic nanodressings are summarized in tabular form in **Tables 4**, **5**.

**Table 4:** Summary of other inorganic nanodressings.

Inorganic nanotype	Mean particle size (nm)	Synthesis method	Carrier	Microbial species affected
Polydopamine-assisted silver nanoparticles (134)	300-500	Chemical reduction	Sericin (SS)/AGAR composite membrane	*E. coli* and *Staphylococcus aureus*
Silver nanoparticles (AgNPs) (135)	35-65	*In situ* synthesis	Polydopamine-coated sericin/polyvinyl alcohol (PVA) composite film	*E. coli* and *Staphylococcus aureus*
Silver nanoparticles (AgNPs) (136)	——	*In situ* synthesis	Sericin/polyvinyl alcohol (PVA) blend film	*E. coli* and *Staphylococcus aureus*
Copper oxide nanoparticles (CuONPs) (137)	88-97	Electrospinning	Polycaprolactone (PCL) film	*Pseudomonas aeruginosa, Klebsiella acidogenes* and *Staphylococcus aureus*
4,6-diamino-2-pyrimidine mercaptan functionalized gold nanoparticles (138)	2.44	Chemical reduction	Fibroin (SF) mixed matrix membrane	MDR *E. coli*
4,6-diamino-2-pyrimidine mercaptan (DAPT) gold nanoparticles (139)	——	——	Bacterial cellulose	*E. coli* and *Pseudomonas aeruginosa*
Zinc oxide nanoparticles (ZnO (NPs) (140)	60-120	Polydopamine (PDA) helps modify	Sericin (SS)/polyvinyl alcohol (PVA)	*E. coli* and *Staphylococcus aureus*
Zinc oxide nanoparticles (ZnO (NPs) (141)	——	Electrospinning technology	Chitosan-polyvinyl alcohol (PVA) nanofibers	*E. coli, Pseudomonas aeruginosa, Bacillus subtilis* and *Staphylococcus aureus*

**Table 5:** Summary of other organic nanodressings.

Active ingredient	Fiber diameter (nm)	Synthesis method	Carrier	Effect
Curcumin (CUR) and tetracycline hydrochloride (TCH) (142)	360-770	Electrospinning technology	Poly-ϵ-caprolactone (PCL)/AV hybrid nanofiber scaffold	Promotes fibroblast proliferation; antibacterial, nontoxic
Aloe vera (AV) (143)	131.6 ± 27.5	Double-nozzle electrospinning technology	Gelatin (gel) and poly (ϵ-caprolactone) (PCL) mixed scaffold	Improves cell activity, sterilizes; nontoxic
Polyurethane and propolis ethanol extract (PU/EEP) (144)	237.3 ± 65.1	Electrospinning technology	Polycaprolactone/gelatin (PCL/gel) nanofiber scaffold	Promotes collagen deposition, inhibits the growth of *Staphylococcus epidermidis*, *Staphylococcus aureus*, and other species
Propolis ethanol extract (EEP) (145)	——	Electrospinning technology	Polyurethane-hyaluronic acid (PU-HA) nanofiber wound dressing	Improves dermal development and collagen deposition; antibacterial
Cinnamon essential oil (CEO) and nano cerium dioxide (nCeO2) (146)	178.5 ± 34.3	Double-spinneret electrospinning technique	Polyurethane (PU) and polyvinyl alcohol-gelatin (PVA/gel) nanofiber scaffolds	Improves cell count; antibacterial
ZM essential oil (147)	218 ± 58	Glutaraldehyde vapor chemical crosslinking	Polyvinyl alcohol-based nanofiber pad	Inhibits the growth of *Staphylococcus aureus, Pseudomonas aeruginosa* and *Candida albicans*

In conclusion, nanomaterials have the following advantages. High surface/volume ratio allows for small filler size and inter-fill distance. Improved mechanical properties, high strength. Resistance to scratches. In addition, metal ion nanomaterials can be repeatedly sterilised to better control wound infection and promote wound healing. However, current nano dressings also have certain shortcomings that need to be further optimized. It still suffers from high resistance to cell infiltration and multiple dressing changes. Insufficient understanding of formulation properties. Structural relationship, need for easier exfoliation of particles, and dispersion. Cost-efficiency (123).

### 5.5 Bioactive dressings

Bioactive materials are biomaterials that cause a specific biological or chemical reaction by the surface of the material that promotes or influences the connection between the tissue and the material, induces cellular activity or regenerates new tissue. Natural biomaterials derived from cells, cytokines, and even plants and their biological derivatives (e.g. exosomes) have particular advantages in biomedical applications. Most of them can, for example, by activating the immune system, also exhibit specific tissue and organ tropisms. And for some living cells (e.g. stem cells) have a strong ability to penetrate tissue and biological barriers. These properties provide an opportunity to construct large molecule drug carriers that can cross physiological barriers and have good efficacy against DFU (148). While smart nanomaterials cause changes in the bacterial cell membrane in wounds by regulating different particle shapes, compositions, sizes and surface charges. It includes compositional changes and reactive oxygen species (ROS) production, lipid peroxidation, loss of respiratory activity, etc. This ultimately allows biofilm disruption and promotes healing of the DFU (149).

We enumerate the use of cells, cytokines, enzymes and inhibitors, outer membrane vesicles, and smart nanomaterials in DFU dressings.

#### 5.5.1 Scaffold dressings with stem cells

Individuals with DFU have usually been in a state of hyperglycemia for a long time, and the affected blood vessels and tissue cells produce different degrees of lesions. A number of animal experiments have shown that stem cell transplantation is effective in promoting hemodynamic reconstruction and regeneration as well as in regulating the secretion of inflammatory factors, growth factors, and immunomodulatory factors. These effects, which are due to the unique paracrine properties of stem cells, give the method great clinical potential for the treatment of DFU. Conventional stem cell transplantation techniques such as systemic intravenous or local intradermal injection have resulted in low cell survival rates. Intravenously injected cells are also rarely effective because they do not target the lesion (150). If stem cells are inoculated into biomaterials such as nanomaterial scaffolds and collagen scaffolds, cell survival and therapeutic potential can be improved, and targeted delivery can be achieved (151). Therefore, the scaffold delivery method plays a key role in determining the efficacy of cell therapies. These material delivery systems can be used to build *in vivo* cell banks that gradually release stem cells that fill defects and participate in the regeneration of vascular networks (152). Overall, stem cells (SCs) have many advantages. It can express many cytokines and a variety of nerve growth factors that modulate immune function in wounds. It can also accelerate DFU healing by promoting angiogenesis, cell proliferation and nerve growth as well as modulating the inflammatory response. SCs are promising for research as they can solve the problem of low stem cell viability and accelerate wound healing by scaffolding drug delivery systems. Many types of SCs are used in the treatment of skin wounds, such as bone marrow mesenchymal SCs (BMMSCs), umbilical cord mesenchymal SCs (UCMSCs), peripheral blood SCs (PBSCs), adipose-derived mesenchymal SCs (AMSCs), placenta-derived mesenchymal SCs (PMSCs), human amniotic fluid-derived stem cells (AFMSCs), and human gingival-derived mesenchymal SCs (GMSCs). Currently, BMMSCs are the most frequently used type (153). These pluripotent stem cells could differentiate into several types of fibroblasts, osteoblasts, chondrocytes, adipocytes, vascular endothelial cells, epithelial cells.

The process by which these useful cells promote DFU healing is also very interesting. Significantly, these cells can promote endogenous angiogenesis through microenvironmental regulation and expression of vascular hemophilic factor (vWF) and vascular endothelial growth factor (VEGF). At the same time, they stimulate epithelial stem cell recruitment through the secretion of tumor necrosis factor-α (TNF-α) and reduce lymphocyte function and interferon gamma (IFN-γ) activity in the inflammatory response (154). Secondly, these cells promote the production of cytokines such as IGF-1, EGF, MMP-2, MMP-9, and the tissue inhibitors of the extracellular receptor kinase (Erk) signaling pathway, metalloproteinase (TIMP)-1 and -2, by human keratinocytes (155). Moreover, they secrete mitogens that stimulate the proliferation of keratin-forming cells, dermal fibroblasts and epithelial cells *in vitro* (156).

Dressings in which stem cells are used as active therapeutic substances have been extensively reported. For example, on the treatment of diabetic rabbit ear ulcers, circulating angiogenic cells (CACs) were isolated from the peripheral blood mononuclear cell fraction. Osteopontin is a stromal cell protein involved in wound healing and acts as a scaffold for the delivery of CACs. This design increases the angiogenic potential of CACs (150). It has also been reported that incorporation of allogeneic nondiabetic bone marrow-derived mesenchymal stromal cells (MSCs) into collagen scaffolds promotes the healing of diabetic rabbit ear ulcers. The efficacy of this dressing is related to the amount of MSCs in the dressing. If a collagen dressing containing 1,000,000 MSCs is used for treatment, a total neovascular length of 270731 ± 146549 mm can be observed. However, collagen dressings containing 100,000 or 50,000 MSCs were used for treatment, and the total length of neovascularization was only 231849 ± 90588mm and 250521 ± 80213mm, respectively. At the same time, the radial diffusion distance of nutrients from capillaries to damaged tissue was significantly shortened to about 5.4 ± 0.7 μm (157). In a study of the tissue-engineered skin substitutes, a three-dimensional bionic scaffold of collagen-chitosan sponge carrying bone marrow-derived mesenchymal stem cells (BM-MSCs) was designed. BM-MSCs secrete collagen and upregulate the expression of proangiogenic factors such as HIF-1α, VEGF and PDGF. These combined effects promoted ulcer healing in diabetic rats (158).

Other stem cell dressings are summarized in tabular form according to the types of delivery scaffolds they employ (**Table 6**).

**Table 6:** Summary of cell dressings created using various delivery scaffolds.

Type of bracket	Type of cell	Animal model	Mechanism of action	Curative effect
Type 1 collagen scaffold (159)	Mouse BM-MSCs and AD-MSCs	Diabetic C57BL/6 mice induced by STZ	Promotes new blood vessel formation and reepithelialization; effectively accelerates wound healing. Notch signaling is upregulated. Increased concentration of macrophages in the wound.	Mouse ADSC can enhance diabetic wound healing, and the therapeutic effect is similar to that of BMSC.
Silk fibroin (SF)/chitosan (CS) scaffold (160)	Rat adipose stem cells (ADSCs)	Stz-induced diabetic Sprague−Dawley rats	Secretes EGF, fibroblast growth factor, insulin-like growth factor and other important cytokines that repair keratinocytes. ADSCs participate in the establishment of a neovascularization bed.	The wound closure rate of treated animals was significantly improved.
Gellan gel - hyaluronic acid (GG-HA) scaffold (161)	Human adipose stem cells (hASC)	Diabetic CD1-ICR mice induced by STZ	Reduces the number of macrophages at the wound site and promotes healing from the inflammatory stage to the proliferative stage. Promotes the re-epithelialization of keratinocytes.	Accelerates wound closure. Increases the thickness of new epidermis.
Type 1 collagen rolling scaffold (162)	MSC of mouse bone marrow origin	Diabetic C57BL/6 mice induced by STZ	The hypoxic core environment of the rolling scaffold activates MSCs to promote cell survival and produce VEGF. Enhances wound angiogenesis.	Cell proliferation increases. Enhanced wound healing.
N-carboxyethyl chitosan and diacylhydrazine adipate crosslinked scaffold with hyaluronate aldehyde (163)	Bone marrow mesenchymal stem cells (BM-MSCs)	Stz-induced SD rats	BM-MSCs secrete growth factors, inhibit the expression of M1 macrophages and promote the expression of M2 macrophages. Promotes granulation tissue formation, collagen deposition, nucleated cell proliferation, and new blood vessel formation.	Promotes diabetic wound healing

SCs express many cytokines and a variety of nerve growth factors and regulate immune function in wounds and may accelerate DFU healing by promoting angiogenesis, cell proliferation and nerve growth as well as modulating inflammatory responses. These investigations have demonstrated that stem cell dressings are unique and that they have better efficacy than other dressings. At this point in time, most stem cell dressings are still being evaluated in animal experiments and have not been directly applied in clinical practice. Research on stem cell dressings has provided clinical experience and potential for the treatment of DFU. It is expected that stem cell dressings will benefit patients in the clinic over time.

#### 5.5.2 Cytokine dressings

Cytokines (CK) are low molecular weight soluble proteins induced by immunogens, mitogens, or other stimulants to be produced by a wide range of cells, and have a variety of functions, including regulation of intrinsic and adaptive immunity, hematopoiesis, cell growth, APSC pluripotency, and repair of damaged tissues. Cytokines suggested to be effective in DFU dressings are Basic Fibroblast Growth Factor (bFGF), Vascular Endothelial Growth Factor (VEGF), and Platelet−Derived Growth Factor (PDGF), among others (164).

Basic fibroblast growth factor (bFGF) can be involved in many biological processes such as angiogenesis, wound healing, neurogenesis, cellular differentiation and migration, and it can bind to all receptors (165). It has been found that the prepared bFGF-gel dressing effectively promotes wound healing in rats. Through histological and immunohistochemical analyses, it was found that bFGF-gel dressing could promote the proliferation of traumatic cells, reduce traumatic inflammation and enhance capillarization (166). It suggests that basic fibroblast factor can be applied to DFU excipients.

The vascular endothelial growth factor (VEGF) family is an important family of growth factors that are key players in the process of angiogenesis. In recent years, VEGF has also been found to have neuroprotective and trophic roles and to be an important signaling molecule for nerve repair and regeneration (167). One study showed that decreased VEGF expression was associated with poor wound healing and an increased ratio of matrix metalloproteinase-9 to tissue inhibitor of metalloproteinase-1 in infected DFUs, thus suggesting that VEGF could be applied to DFU dressing disease to promote wound healing (168).

#### 5.5.3 Exosomes dressings

Exosomes are nanoscale lipid bilayer-enclosed structures carrying proteins, lipids, RNAs, metabolites, growth factors, and cytokines that can play key roles in mediating intercellular communication both locally and systemically (169). A study showed that the application of autologous mesenchymal stem cell exosomes to treat high glucose-induced HUVECs or DFU mice revealed that mmu_circ_0001052, an exosome of Adipose-derived stem cells (ADSC), had a better effect in promoting wound healing and improving wound area. And the mechanism of action of mmu_circ_0001052-miR-106a-5p-FGF4 mRNA network in DFU angiogenesis was verified (170). Another study showed that exosomes isolated from platelet-rich plasma (PRP-exos) had a promising therapeutic effect on DFU wounds and verified the involvement of MALAT1-mediated signaling in the treatment of DFU wound healing by PRP-exos. This may help to identify the best targets and effective therapies for DFU treatment (171).

In conclusion, exosomes have a high targeting capacity, which improves the efficiency of drug use and reduces the frequency of drug use. It also has the advantages of high drug-carrying capacity and high loading efficiency. And it can promote low immunogenicity and reduce body clearance. It has high temporal stability and can produce combined and synergistic therapeutic effects (172).

#### 5.5.4 Autologous platelet-rich plasma dressings

In recent years, an increasing number of studies have demonstrated the unique clinical advantages of autologous platelet-rich plasma (PRP) dressings (173–175). It has been confirmed that autologous platelets are enriched with more than 1100 different protein types and contain more than 1500 protein-based bioactive factors (176). The most abundant proteins in platelets are signaling proteins, including growth factors (epidermal growth factor (EGF), vascular endothelial growth factor (VEGF), transforming growth factor-β (TGF-β), insulin-like growth factor-1 (IGF-1), chemokines and other cytokines (interleukin-1β, platelet basic protein, platelet factor 4, and C-C chemokine ligand 5), adhesion proteins (vitamin d-binding proteins, fibrinogen, fibrinogen, fibronectin, and vitreous connecting proteins), proteases, and antiproteases (177). On the other hand, platelets contain amino acids, hormones (insulin, estradiol, adrenocorticotropic hormone, androgens, estrogen, progesterone, and human growth hormone), corticosteroids, thyroxine, serotonin, adrenaline, histamine, enzymes, vitamins, organic acids, pigments, ions, dissolved gases, nutrient molecules, and metabolic products (178). Wound healing can be accelerated and supplied with substances through Autologous platelet-rich plasma dressings due to the many active ingredients enriched in platelets.

In one study, 90 patients with DFU were randomly divided into a local injection of PRP supplemented by hydrogel coverage group (Group A), a PRP gel and hydrogel dressing coverage wound group (Group B), and a hydrogel dressing covering wound group (Group C). The wound healing rate in Group A was 93.2% ± 0.8%, approximately 41.1% and 71.9% higher than the healing rates in Group B and Group C, respectively. The mean duration of hospitalization for Group A patients was 40.5 ± 1.8 days, approximately 21 days and 48 days shorter than those of Groups B and C, respectively. There were significant differences both in wound healing rate and in duration of hospitalization (179). The most important mechanism responsible of PRP dressings is that these dressings release growth factors in proportions that optimally promote gene expression in target cells. Thus, they increase collagen synthesis and promote cell division and proliferation. In addition, because white blood cells and platelets have similar sedimentation rates, PRP obtained by centrifugation contains a certain concentration of white blood cells, improving its local anti-infection ability. Since PRP is extracted from the patient, it is low in immunogenicity and high in safety (180). At the same time, it has also been reported that PRP can be uniformly incorporated directly into collagen-glycosaminoglycan (collagen-gag) scaffolds. This loaded scaffold releases key growth factors that promote wound healing. It can be used to overcome the bottleneck created by collagen-gag scaffolds that rely only on local endogenous signals to promote healing (181). For the reasons discussed above, PRP dressing therapy is widely popular in the clinic and can greatly reduce the long-term medical burden of patients with DFU.

Platelets release growth factors, cytokines and interleukins, which have a critical impact on healing mechanisms, including angiogenesis, cell migration and proliferation and ECM protein synthesis (182). The efficacy of Autologous platelet-rich plasma dressings appears to cover a wide range of indications. The use of autologous PRP improved wound healing in a shorter period of time compared to traditional wound care. Platelet-rich plasma may be an effective and promising treatment for chronic DFU, with PRP being able to heal in a shorter period of time. However, the mechanism of action of these products has not been fully elucidated.

#### 5.5.5 Acellular wound matrix

Decellularized extracellular matrix (dECM) is obtained from human or fish skin by decellularization technologies that include chemical methods, physical methods, enzymatic treatment, and osmotic treatment (183–186). Unlike the aforementioned collagen dressings, dECM contains approximately 75% natural collagen but also includes fibrin, fibritin, proteoglycans, glycosaminoglycans, stromal cell protein, and other proteins (187, 188). Current studies have shown that dECM not only anchors cells but also has activities that affect cell survival, proliferation and function. Various components of dECM with specific functions interact with each other to promote wound healing (188). Decellularized fish skin matrix is rich in a large number of lipids that are omega-3 fatty acids, especially eicosapentaenoic acid (EPA) and docosahexaenoic acid (DHA). These compounds regulate wound healing processes, form bacterial defense barriers, and alter skin physiology at the cellular and molecular levels (189). Another advantage of using decellularized matrix therapy in cases of dermal trauma is that dECM is almost cell-free and weakly immunogenic. The ECM is a major component of the skin and is critical for chronic wound healing. Thus, dECM is an emerging research target for the clinical application of bioactive dressings. A randomized clinical trial showed that wound dressings containing human decellularized dermal matrix (ADM) exhibited a trend toward better wound healing and greater wound area reduction compared to conventional care in a controlled trial involving 168 DFU patients (190).

ECM compositions are emerging bioactive wound dressings due to their ability to modify cellular properties in healing wounds. Despite the excellent biological properties of conventional ECM membranes and their demonstrated efficiency in the clinical treatment of skin wounds, there are still some drawbacks that prevent their widespread use. Considering that most ECM membranes do not possess antimicrobial properties, the risk of potentially transmitting fungal, bacterial, or viral infections should be carefully addressed to avoid any unfavourable complications. In addition, due to the heterogeneity of biologically derived materials, the development of standard protocols to improve the consistency of ECM membranes is necessary for future clinical applications.

#### 5.5.6 Smart nanomaterials dressings

Smart polymer nanomaterials are able to dynamically sense changes in environmental stimuli and respond accordingly by changing their physicochemical properties, similar to the self-regulation and adaptive ability of biological systems in nature (191). If the molecular structure is applied to the diabetic foot ulcer dressing after careful design, the dressing can respond to a variety of stimuli such as changes in ambient temperature, pH, light, ions, molecules, electric and acoustic fields, which is more conducive to the healing of DFU wounds. The most introduced smart nanomaterials are nanoemulsions and nanoparticles.

Nanoemulsions are kinetically stabilized emulsions with nanoscale droplet sizes (192). It is a widely used formulation in diabetic wound healing applications due to its excellent physicochemical properties and high patient tolerability. It was found that the synergistic effect of insulin-loaded nanoemulsion and homogenized aloe vera gel given to diabetic rats resulted in faster wound closure (193). And it proved to be an effective and promising treatment for diabetic wounds. A naringenin nanoemulsion gel enriched with tocotrienols has been formulated for the treatment of diabetic foot ulcer wounds. The droplet size, surface charge, spreadability, polydispersity index, viscosity, *in vitro* release kinetics and mucosal adhesion properties of the stabilized nanoemulsion gel were evaluated by several metrics. The results showed that an increase in polymer concentration of the nanoemulsion gel increased the mucosal adhesion properties and decreased the drug release rate (194). Thus, the use of nanoemulsion gels is a promising approach to wound management associated with diabetic complications.

Nanoparticles with small size and large surface area to volume ratio are effective in increasing penetration and biological interactions at the wound site. It triggers cell proliferation, cell signalling, cell-cell interactions, vascularisation and epithelialization (195). Therefore, it is ideal for topical drug delivery applications. It has been reported that gelatin nanoparticles were constructed to test the therapeutic effect on diabetic foot ulcers by *in vitro* model human endothelial cells and *in vivo* model diabetic foot ulcer rats. It was found that the nanoparticles showed higher wound healing rate, cell proliferation, blood vessel formation and epithelialization (196).

In summary, nanomaterials, especially smart nanomaterials, have outstanding performance and great research prospects in diabetic foot ulcer treatment. In the future, smart nanomaterials will appear in diabetic foot ulcer dressings with outstanding performance.

### 5.6 Dressings and modern technology

Current academic research on the development of dressings for chronic wounds is not limited to the simple mixing of various biological materials. Current designs are more individualized and are based on the wounds of the patient. Dressings that are based on the specific wound morphology and the condition of the lesion eliminate the mismatch between the wound and the dressing size and improve the patient’s fitness. Moreover, this multidisciplinary approach integrates physics, zoology, and intelligent technology. Functions such as real-time dynamic monitoring and wound response can be added to the treatment.

#### 5.6.1 3D bioprinting technology

3D printing (3DP) is a technology that uses a digital model file as the basis for constructing an object by printing layer by layer using a bondable material such as powdered metal or plastic. For the medical field, it is undoubtedly a great boon. the maturity of 3DP technology has largely inspired the rapid development of reconstructive bionics. Especially for chronic wounds such as DFU, its emergence has given hope to diabetic foot ulcer patients. Currently, the most established 3DP technology is Drop-on-demand (DOD), which offers the advantages of low cost, fast printing speed, high resolution, and the ability to change the concentration gradient (197). However, there are drawbacks such as low inoculum density and impaired cell viability and function due to cross-linking and gelation processes. The study reports the use of 3D bioprinting to fabricate implantable multilayer vascularized bioengineered skin grafts. The graft is formed using one bioink containing human foreskin dermal fibroblasts (FBs), human endothelial cells (ECs) derived from cord blood human endothelial colony-forming cells (HECFCs), and human placental pericytes (PCs) suspended in rat tail type I collagen to form a dermis followed by printing with a second bioink containing human foreskin keratinocytes (KCs) to form an epidermis. *In vitro*, it has biological and morphological functions comparable to those of natural human skin (198). Provide solid evidence for the use of 3DP technology in DFU. The current research hotspot is more inclined on how to design innovative, individualized and versatile 3DP technology and apply it with diabetic foot ulcer wounds. For innovative technologies, the design of novel 3D printed biomaterials with mechanical, rheological and biological properties that match those of the target tissue is a key factor. In the case of individualized techniques, each patient’s condition and physical functioning is different. In the future, precision medicine will be a big trend. The 3D bioprinting technology converts the raw material for preparing a variety of dressings into a bio-ink, which can then quickly seal skin defects according to the contours of the wound. Specifically, when diabetic foot ulcers occur, the wound site is scanned to prepare an accurate 3D model for 3D printing. Once the 3D model is obtained, it is transferred to a printer with the corresponding bioink and converted to a 3D printed toolpath. The printed scaffold is then crosslinked and applied to the wound site. The design of personalized adjuncts based on the size and shape of the wound in diabetic foot ulcer patients adapts to the patient’s unique wound topology to ensure complete wound coverage and better aesthetics after healing (199, 200).

Acellular dermal matrix (ADM) and gelatin methacrylamide (GelMA) bioinks with shear-thinning properties print simulated full-layered skin. This not only enhances cell viability and proliferation but also supports *in vitro* epidermal reconstruction and improves wound healing quality (201). Another report describes a digital light processing (DLP)-based 3D printing technique that prints functional living skin (FLS). It used gelatin methacrylate (GelMA), hyaluronic acid (HA-NB), and photoinitiator phenyl lithium-2,4,6-trimethylbenzoyl phosphite (LAP) as bioink. This method allows precise targeting of human skin fibroblast (HSF) and human umbilical vein endothelial cell (HUVEC) clusters with high cell viability and thereby promotes skin regeneration and neointima formation (202). Research has designed a biomaterial that can be 3D printed. It contains functionalized sodium alginate (FSA), biomineralized silica, and DNA from salmon sperm. And investigated the chronic wound healing ability of DNA-bSi30@FSA dressings in mouse models of diabetes. On the 6th day of local wound monitoring, the residual wound in the DNA-bSi30@FSA dressing group was significantly reduced (50.5%). The wound area in the control group, FSA, DNA@FSA and Si30@FSA dressing groups was still 89.7%, 87.3%, 66.8% and 61.9%, respectively. Finally, on day 15, the wounds treated with the DNA‐bSi30@FSA, Si30@FSA, and DNA@FSA dressing groups showed faster healing than those of the saline and FSA dressing groups. Thus, the 3D‐printed DNA‐bSi30@FSA dressing could significantly enhance wound healing in a chronic wound in diabetic mice by enhancing the synergistic bioactive functions of DNA and biomineralized silica nanotherapeutics (203).

3D oprinting has emerged as a promising technology designed to rapidly close skin defects according to their contours. The 3D bioprinted skin substitute has a strictly layered structure with controlled cell type and density localisation, enhancing homology with natural human skin. It also offers better cost and time efficiency. However, 3D bioprinting still has some limitations and requires long-term evaluation studies in large animal models to confirm its future clinical potential. Its precise molecular mechanisms have not yet been elucidated.

#### 5.6.2 Light, heat and electrical effects

Scholars have focused considerable attention on the auxiliary effects of light, heat, and electricity in dressing applications in recent years (204). Multicolor light irradiation in the near infrared region (NIR) is most commonly reported. Physical stimulation and photoactivation can increase the biological effects of a variety of materials (205). Photothermal therapy (PTT) mainly destroys bacterial cell membranes and biofilms by light-induced heat generation. NIR laser irradiation also has a bactericidal effect through its effects on ROS levels, ATP levels, lipid peroxidation, glutathione and adenosine triphosphate accumulation, and bacterial membrane disruption; through these mechanisms, it appears to assist in eradicating multidrug resistant bacteria and accelerating wound healing in MRSA-infected diabetic models (206–208). In DFU treatment, PTT can be combined with chemobacteriological therapy to form a synergistic antibacterial strategy. At present, metal nanoparticles, non-metallic nanoparticles, organic dyes, etc. have been found to be used as photothermal conversion agents. Among them, black phosphorus (BP) showed high photothermal conversion efficiency. In one study, BP modified with bismuth oxide (Bi_2_O_3_) and ϵ-polylysine (ϵ-PL) was reported. When BP/Bi_2_O_3_/ϵ-PL is infiltrated into the hydrogel, NPs@gel-2 is obtained. NIR irradiation triggers the photothermal conversion capability of BP/Bi_2_O_3_. ϵ-PL generates high temperatures to further damage bacterial cell membranes and lead to leakage of intracellular substances, achieving sterilization and preventing biofilm formation. In the *in vitro* antimicrobial test, NPs@gel-2+NIR was 100% inhibited against *Pseudomonas aeruginosa*, *Staphylococcus aureus* and *Escherichia coli*. And on day 14 of the infected wound model monitoring in diabetic animals, the wound shrinkage rates of each group are sorted as follows: NPs@gel-2+NIR (98.8%) > Control (−) (refers to an uninfected wound) (94.2%) > NPs@gel-2 (88.1%) > Blank gel (81.2%) > Control (+) (71.7%) (209).

After the wound appears, the movement of ions begins to repair the wound and create an endogenous electrodynamic field. Endogenous and exogenous electric fields can provide the earliest signals needed to initiate cell proliferation, migration, and eventual wound epithelialization. Changes in the electric field then direct cells, molecules, and drive the wound healing process. The final charge and bioelectric dynamic field penetrates into several stages of wound healing, driving cells and molecules and maintaining the flow of oxygen and nutrients necessary for wound healing. Many treatments can promote wound healing by influencing electrical factors. For example, exogenous electric fields such as pulsed electromagnetic fields (PEMF), pulsed high-voltage stimulation (PHVS), and low-level laser therapy (LLLT) promote wound healing. LLLT can produce electrical action because it increases the yield of ATP, thereby improving the efficiency of the sodium-potassium pump. The potential difference between the inside and outside of the battery is guaranteed (210). Microfabricated electrodes, pH-sensitive hydrogels, and controlled electronic circuits can be added to dressings. And the release of the drug by applying a voltage to change the pH near the electrode. This results in a dressing that allows flexible stimulus-response drug delivery (211). Therefore, not only can an electrical stimulus be applied to the dressing, a low voltage can also be applied directly to the wound, providing a new treatment that accelerates wound healing. Electrodynamic fields direct the migration of fibroblasts, keratinocytes, macrophages, and epithelial cells and influence blood rheology and microcirculation to promote wound healing. For example, microbattery-impregnated bioelectric dressings (BEDS) allow an animal’s wound to close completely within 4 weeks without infection or transplantation. Bioelectric dressings are therefore a promising wound dressing for DFU (210, 212). In addition, a pulsed capacitive coupled electric field (PCCEF) platform has been researched and developed. When the pulse width ≥ 10 μs, PCCEF significantly promoted the migration and proliferation of human dermal fibroblasts and HaCaT cells, enhanced M2-type polarization of macrophages, and promoted wound healing in mouse models (213).

Light, heat and electricity are excellent aids in dressing application. Physical stimulation and photoactivation can enhance the biological effects of a wide range of materials. Light stimulation of platelets has great potential for platelet activation and fibroblast stimulation. The electric field directs the migration of fibroblasts, keratinocytes, macrophages and epithelial cells, affecting blood rheology and microcirculation, thereby promoting wound healing. However, relevant studies are currently inadequate, limiting its widespread clinical use.

#### 5.6.3 Microneedling dressings

A painless and simple drug delivery system known as microneedling (MN) has been developed since the turn of the 21st century. The MNs used in this system contain porous structures with continuous nanometer- or micron-scale pores that transport drugs or biofluids through capillary action. Changing the porosity of these structures affects the internal fluid flow, and this in turn adjusts the mechanical strength of the MN device (214). The stratum corneum (SC) is the outermost keratinizing layer of the skin, and only molecules smaller than or equal to 500 Da (dalton) in size can move freely in the skin. Microneedles can create microchannels through the SC of the skin without stimulating proprioceptive pain nerves (215). And there are numerous studies showing that MNs can successfully deliver both small and large molecule drugs (e.g., insulin, vaccines, proteins, and chemotherapeutic agents) through the skin. Compared with conventional bandages and hydrogels, MNs have the advantage of transporting drugs through deeper layers of skin and improving drug delivery efficiency (216). The chances of infection when using MN are much smaller than with traditional hypodermic needles. There is great interest in the development of MN dressings for DFU. Inspired by the structure of mosquito mouthparts, MN devices with fixed and liquid transfer parts have been developed. In addition, the dressing as a whole features an ultrafine needle tip, a personalized pattern design, and programmable needle length and can be prepared with a variety of mechanical strengths to realize intelligent painlessness (217). Inspired by the flat and sloping structure of shark teeth, MN patches are designed to provide stable adhesion. MN can also be combined with MXene electronics to provide sensitive monitoring of the motion of the dressing (218). Inspired by the highly folded structure of insect wings, the versatile three-dimensional (3D) origami MN patch features an ultrafine needle structure, microfluidic channels, and circuits. It promotes wound healing by releasing drugs in a controlled manner and monitoring exercise (219). In one study, a near-infrared (NIR)-responsive hair microneedle patch was reported. It contains hierarchical microparticle (HMP), ZnO, vascular endothelial growth factor and basic fibroblast growth factor. It delivers drugs to the extremities painlessly, accurately and controllably under NIR irradiation. Among them, hair-derived HMP exhibits the ability to clear ROS, thereby preventing damage to blood vessels. At the same time, zinc oxide (ZnO) nanoparticles confer excellent antibacterial activity on the MN patch, and the photothermal effect of HMP under near-infrared radiation can further enhance this activity. *In vivo*, it significantly raises the temperature of the fingertips of diabetic rats and promotes collagen deposition and angiogenesis during wound healing (220). In addition, the development of hydrogel dressings in the form of microneedles exhibits better sustained release of drugs, adequate mechanical properties, and better biocompatibility than traditional dressings (221, 222).

Microneedling can safely and sustainably deliver large amounts of therapeutic agents through the skin without compromising painless injections. And does not increase the risk of infection. Microneedle dressings accelerate the healing process of diabetic wounds, reduce the inflammatory response, promote collagen deposition at regenerated tissue sites, and improve glycaemic control in animals. However, once the microneedle dressing adheres to the skin, it is difficult to peel off from the skin. And there are still individual differences in side effects such as skin redness, irritation, or skin allergies. If high doses are required for treatment, the MN patch may be underloaded, so the MN patch must be used multiple times. It is effective in diabetic wound management and has great potential in the treatment of other chronic skin injuries.

#### 5.6.4 Intelligent bandages

The smart bandage is a product of wearable technology for the treatment of DFU. With the development of the Internet of Things, and emerging biomaterials, wearable sensing and information and communication technologies are key steps in driving the transformation of health care services to a new model of connected health (CH) care (223). In the clinical diagnosis and treatment of DFU, the healing stage of the wound and the existence of complications such as infection are usually judged only by medical evaluation and by the naked eye. The use of such rough wound assessment and fixed dressing change patterns not only frequently results in missing of the optimal treatment time but also leads to unnecessary dressing changes and increased medical costs. Smart bandages solve this problem. Smart bandages based on wearable technology are mainly used for integrated wound identification, real-time dynamic monitoring of wounds in which information on important parameters is collected, and early prediction of infection. In addition to 3D printing, online wound image scanning and recognition technologies such as image recognition, computer modeling, nanomaterial fabrication and modification, combined with offline smart material manufacturing, can further promote the individualized design of wound dressings (224). Smart bandages monitor pH, sodium, potassium, calcium and uric acid levels, and wound temperature in real time to provide quantitative diagnosis (225). The basic principle on which they work is that the wound exudes fluid into the sensing area or excites the pH response current, resulting in flow analysis results through voltage changes and potential conversions (226, 227). In a pioneering study, a flexible bioelectronic system was developed. It facilitates the integration of current smart bandage technology with sensors and stimulators. This system consisting of wirelessly powered, closed-loop sensing and stimulation circuits with skin-interfacing hydrogel electrodes capable of on-demand adhesion and detachment. The system continuously monitors skin impedance and temperature and provides electrical stimulation depending on the wound environment. Across preclinical wound models, the treatment group healed ~25% more rapidly and with ~50% enhancement in dermal remodeling compared with control (228). In addition, a smart disinfection bandage based on wirelessly powered ultraviolet C (UVC) radiation has been reported. The induction coil is seamlessly hidden in a fabric bandage and coupled to the rectifier circuit. This system can effectively eradicate Gram-negative bacteria and *Pseudoalteromonas sp* (229). Nowadays, a wide variety of mobile applications are widely used worldwide in many areas of daily activities, which greatly improve the quality of human life. Meanwhile, mobile applications for DFU monitoring and care are being developed. Cassidy et al. developed the first mobile app capable of accurate DFU detection using AI and cloud-based technologies. This system was tested in a 6-mo clinical evaluation at two UK National Health Service hospital sites (Lancashire Teaching Hospitals and Salford Royal Hospital) and is currently being further developed to improve functionality and accuracy (230). The success of this type of program development also provides some guidance in the selection of dressings.

#### 5.6.5 Orthopedic prosthetics and regenerative medicine

An orthopedic prosthesis is a medical device designed to replace missing or damaged bones and joints, thereby restoring mobility and function to individuals with musculoskeletal injuries or conditions. But improving the biocompatibility of orthopedic prostheses to promote better integration with natural tissues is an urgent problem. Regenerative medicine focuses on how to induce human tissue regeneration and identify instructive cues that direct refractory tissues down a regenerative path (231). This suggests the potential of regenerative medicine to use natural tissue repair and regeneration to improve the biocompatibility of prostheses and potentially replace lost tissue. Cells, growth factors, and biomaterials can be used to stimulate the body’s natural regenerative ability to repair damaged tissue. Therefore, by using regenerative medicine techniques, we can develop orthopedic prostheses that are more compatible with natural tissues. Its application to ulcer defects in DFU patients is expected to reduce the risk of various complications (such as infection, inflammation, rejection) and improve long-term outcomes for patients. In addition, as mentioned earlier, patients with DFU have a high rate of amputation. For these patients, orthopedic prostheses are undoubtedly a huge boon. Sensory neuroprosthetic devices have been designed to provide individuals with the sensation of natural feet, enabling them to walk more confidently and controllably (232).

## 6 Healing of diabetic foot ulcers

**Figure 4:**
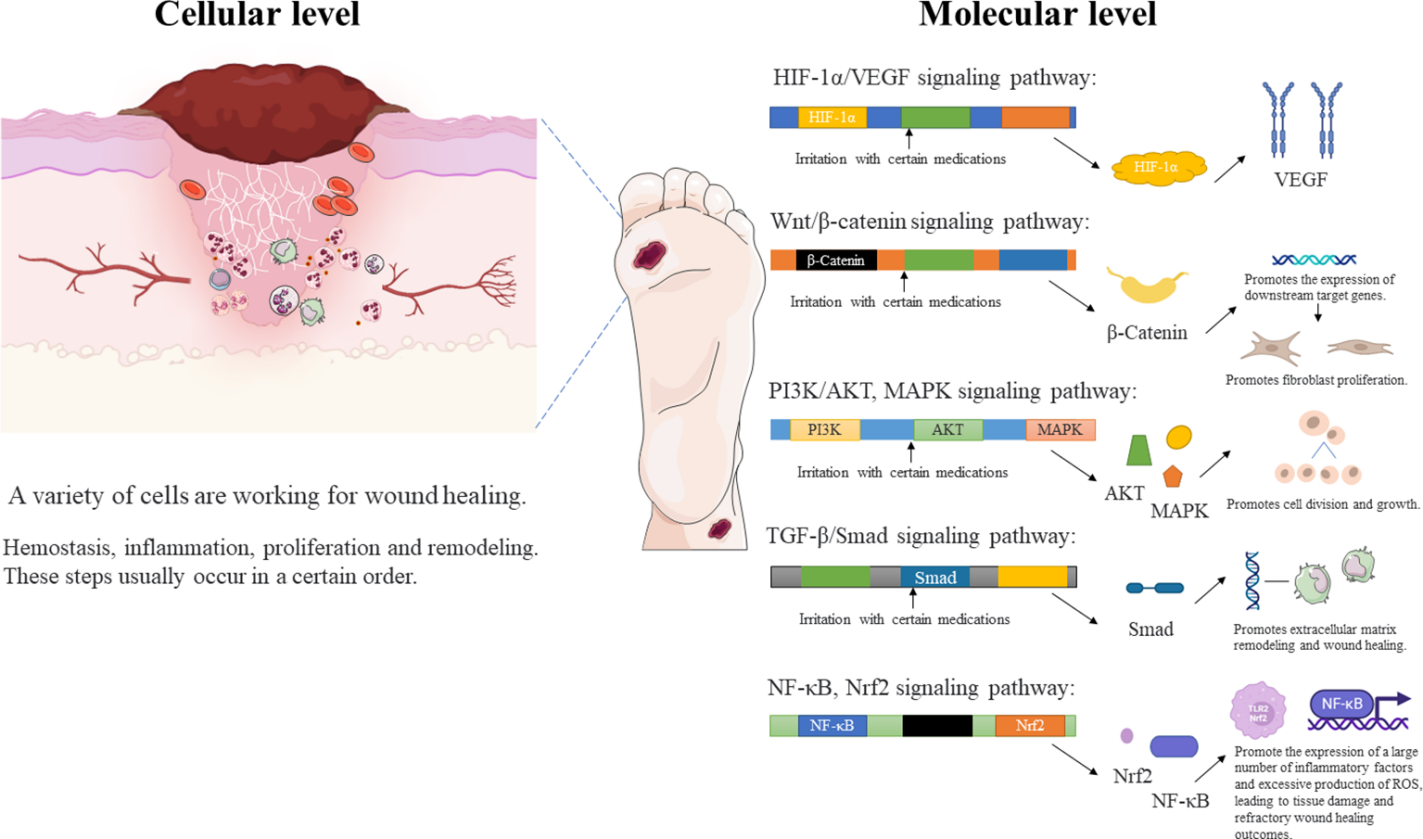
The healing mechanism of DFU.

The healing of DFU is complicated. At the cellular level, it is the result of multiple cells working together. At the molecular level, it can affect the activities of various cell types through the activation of many signaling pathways. With continuous improvements in science and technology, the healing process of DFU is gradually becoming clear, and this has a very significant effect on clinical treatment (**Figure 4**).

### 6.1 Diabetic foot ulcer healing at the cellular level

Wound healing is normally a dynamic process. It occurs in four main stages: hemostasis, inflammation, proliferation and remodeling. These stages usually occur in a specific order. Hemostasis occurs immediately after injury; it is characterized by recruitment of platelets and circulating clotting factors to the wound site to initiate clotting. When platelet recruitment occurs, damaged cells release signaling factors that activate resident macrophages and damage-related molecular patterns. At the same time, stimulated polymorphonuclear neutrophils (PMNS) enter from the vasculature to defend against pathogens. When PMNs begin to migrate to the wound, they initiate the inflammatory phase. Neutrophils release chemokines that recruit circulating monocytes from the peripheral blood to the wound site. The recruited monocytes differentiate into macrophages and dendritic cells. They perform key steps in the inflammatory phase of wound healing. The proliferative stage begins with the recruitment and activation of keratinocytes and fibroblasts. At this stage, growth factors stimulate keratinocytes to re-epithelialize the wound. During this time, the temporary matrix established by platelets during hemostasis is replaced by granulated tissue. Fibroblasts secrete proteases and matrix metalloproteinases (MMPs) that degrade the temporary matrix. They also secrete collagen and other extracellular matrix (ECM) proteins into the granulation tissue. The final phase, the remodeling phase, begins as soon as granulation tissue appears. Here, fibroblasts differentiate into wound contraction myoblasts, and the collagen III that was deposited in the ECM during the proliferation stage is exchanged for collagen I, which has greater tensile strength (233, 234).

### 6.2 Diabetic foot ulcer healing at the molecular level

#### 6.2.1 HIF-1α/VEGF signaling pathway

Vascular endothelial growth factor (VEGF) is a highly specific endothelial growth factor. It can promote increases in vascular permeability, extracellular matrix degeneration, vascular endothelial cell migration, proliferation and angiogenesis. Serum levels of miR-217, HIF-1α, and VEGF were measured in patients with DFU, in patients with simple diabetes mellitus (DM), and in healthy controls. Rat models of DFU were also established and treated with miR-217 inhibitors and/or with HIF-1α siRNA. It was found that inhibition of miR-217 upregulated the HIF-1α/VEGF pathway, promoted angiogenesis and decreased inflammation in DFU rats, thus effectively promoting healing of ulcer sites (235). Zhu et al. confirmed that activation of the HIF-1α/VEGF/VEGFR2 pathway promotes angiogenesis and showed that increasing angiogenesis has a therapeutic effect on wound healing in DFU (236).

#### 6.2.2 Wnt/β-catenin signaling pathway

β-catenin is an important downstream factor in the Wnt pathway and is a multifunctional protein. It is closely related to skin damage and healing. When the Wnt/β-catenin pathway is activated, phosphorylation of β-catenin in the cytoplasm is inhibited, degradation is reduced, and β-catenin accumulates continuously. When the amount of β-catenin reaches a certain level, it enters the nucleus and interacts with T-cell transcription factors and lymphoid-enhancing transcription factors to form protein complexes. In this way, it can promote the expression of downstream target genes, facilitate the generation of epidermis and keratinocytes, and promote wound healing (237). *Panax notoginseng* has been used to treat diabetic models. It was found that PN improves albuminuria and podocyte EMT in diabetic rats by inhibiting the Wnt/β-catenin signaling pathway, providing experimental support for novel treatment options for diabetic neuropathy (238).

#### 6.2.3 PI3K/AKT signaling pathway

Protein kinase B, Akt, also known as PKB or Rac, plays an important role in cell survival and apoptosis. The PI3K/AKT signaling pathway regulates many critical cellular processes, including nutrient uptake, anabolic response, cell growth, differentiation and survival, proliferation, and cell motility (239). It was found that when the miR-138 inhibitors IGF-1 and LY294002 were administered to DFU rat models, the resulting downregulation of miR-138 alleviated the animals’ inflammatory responses and promoted healing of DFU by stimulating the PI3K/AKT pathway and hTERT (240). Use of the plasma ED-EV method to treat diabetic mice has also been described in the literature, and it has been confirmed that this method of treatment slows the aging of mouse fibroblasts and accelerates wound healing by promoting YAP nuclear translocation and activating the PI3K/Akt/mTOR pathway (241).

#### 6.2.4 TGF-β/Smad signaling pathway

Transforming growth factor-β (TGF-β) is considered to a polymorphic signaling pathway that is involved in many processes in both mature organisms and developing embryos, including cell growth, differentiation, apoptosis, the epithelial-mesenchymal transition, and extracellular matrix production. Smad proteins act downstream of the TGF-β family of receptors and carry signals from the cytoplasm to the nucleus resulting from the binding of TGF-β and its receptors. The TGF-β/Smad signaling pathway plays a key role in regulating extracellular matrix remodeling and wound healing (242). By observing wound healing in DFU mouse models, researchers found that the number of WDR74 and M2 macrophages in the wound tissue of DFU mice was decreased. Activation of the TGF-β/Smad pathway increased the expression of M2 macrophage markers (argininase-1 and YM1) and IL-4 while decreasing the expression of M1 macrophage markers. TGF-β/Smad pathway activation also promoted ECM production and facilitated wound closure in diabetic mice. Overexpression of WDR74 increased Smad2/3 phosphorylation, increased the number of M2 macrophages and the production of ECM, and alleviated DFU (243).

#### 6.2.5 MAPK signaling pathway

Mitogen-activated protein kinases (MAPKs) are a group of evolutionarily conserved serine/threonine protein kinases. They are involved in various biological processes such as cell growth, apoptosis, hormone signaling, the immune response, and the inflammatory response. MAPK genes can be divided into three main subfamilies, namely, extracellular signal-regulated kinases (ERKs), Jun N-terminal kinases (JNKs) and p38 MAPKs (244). Zhu et al. used bioinformatics methods to screen for novel genes that play an active role in diabetes-related fibroblasts. The results showed that the MAPK signaling pathway plays a key role in the regulation of diabetic wound healing. MAPKAPK3, HSPA2 and TGFBR1 are potential key genes in this regulatory process. ETV4 and NPE2 play a potential role in the regulation of wound regeneration in DFU (245).

#### 6.2.6 NF-κB signaling pathway

Nuclear factor-κB (NF-κB) is an important cellular kernel transcription factor that is involved in many physiological and pathological processes, such as inflammatory responses, immune responses, cell survival, and apoptosis. The NF-κB pathway is the most typical proinflammatory signaling pathway because it is activated to express a large number of proinflammatory factors, including cytokines, chemokines and adhesion molecules (246). Sun et al. treated a rat model of diabetic foot ulcers with paeoniflorin and found that paeoniflorin effectively inhibited NLRP3- and NF-κB-mediated inflammation in DFU by inhibiting CXCR2. Wound inflammation in DFU rats was greatly reduced, and wound healing improved (247).

#### 6.2.7 Nrf2 signaling pathway

Nuclear transcription factor-erythroid 2-related factor 2 (Nrf2) belongs to the Cap-n-Collar family of alkaline leucine zipper proteins and is part of the most significant antioxidant stress signaling pathway. The imbalance of free radicals and antioxidants that occurs in DFU patients may lead to excessive production of ROS, resulting in tissue damage and refractory wound healing (248). Sun et al. established streptozotocin (STZ)-induced diabetic rat models and human immortalized keratinocytes treated with high glucose (HG). Both models were treated with paeoniflorin. It was found that STZ-induced diabetic rats had delayed wound healing compared with normal rats. The animals are characterized by severe oxidative DNA damage, low expression of vascular endothelial growth factor (VEGF) and transforming growth factor β1 (TGF-β1), and increased apoptosis. Treatment with PF activated the expression of Nrf2 and improved wound healing in DFU rats. *In vitro* experiments have also shown that PF accelerates wound healing, alleviates oxidative stress, increases cell proliferation and migration, reduces apoptosis, and increases the expression of VEGF and TGF-β1 through the Nrf2 pathway under hyperglycemic conditions (249).

## 7 Perspectives

This review summarizes the properties of different dressings to help healthcare professionals better select dressings, summarizes the healing mechanisms of diabetic foot ulcers at the cellular and molecular levels, and serves as a reference for researchers trying to develop dressings that target specific mechanisms. DFU is a devastating complication of diabetes mellitus associated with infection, amputation and death and are affecting an increasing number of diabetic patients. Dressings play a very important role in the management of DFU, and different categories of dressings each have their own advantages and disadvantages. The correct use of dressings can improve the healing rate of DFU and lower the cost of treating DFU. However, due to the complex pathogenesis of DFU, susceptibility to infection, long duration of the disease, and the possibility of recurrence, treating DFU is a major challenge for physicians and patients. Currently, there are some challenges and limitations regarding the research and application of dressings.

At the laboratory stage, healing of rat skin wounds is very different from that of mouse wound healing models compared to human wound healing, and some of the available experimental data have been obtained from small randomized controlled trials with a high risk of bias. In addition, due to the complex pathogenesis of DFU, it is difficult to understand how certain dressings promote skin regeneration and how they interact with wound tissue cells.

At the clinical trial stage, many dressings are in dire need of well-designed randomized controlled trials to validate efficacy, and robust clinical trials are lacking. Despite the complexity and high-hazard nature of DFU, clinical trial research has accelerated the development of ideal dressings that offer hope to DFU patients. We should pay attention to promote the clinical application of emerging dressings to truly benefit patients.

At the stage of clinical application, at present, the types of clinically applied dressings are still relatively small, and they need to be changed frequently, and the replacement process consumes a lot of manpower, material and financial resources, and the effect is poor, which consumes the energy and confidence of doctors and patients. Poor patient compliance, the price of dressings is too high will also affect the clinical application of dressings.

For patients, inexpensive dressings with better efficacy, fewer potential complications, and the ability to reduce pain are more likely to be accepted.

For physicians, the quality of wound care depends largely on the correct choice of dressing. This requires medical staff to have a good understanding of the properties of different dressings to select the right dressing and change it regularly. The selection of wound dressings should be based on the specific conditions of the patient and the unique advantages of the dressing to maximize the benefits to the patient, so that the individual application of the dressing can be achieved. However, there is no standardized set of guidelines for dressing selection that can be referred to.

DFU is a prevalent and serious global health problem, suggesting future research into higher-quality clinical dressings and a more comprehensive and systematic evaluation of the effectiveness of dressings. Current dressings have their limitations, and research into the “ideal” multifunctional dressing could benefit patients with DFU. The ideal dressing should have good moisture balance, protease barrier, growth factor stimulation, antimicrobial activity, oxygen permeability, and the ability to promote autolytic debridement. Based on the recognition of the above issues, the future development of dressings should focus on intelligence, personalization, multi-target coverage, combined application of multiple dressings and accelerated clinical translation. Current research on dressings in DFU management lacks clear evidence-based guidelines and robust clinical trials on their effectiveness. There is no standardized set of guidelines for dressing selection that can be referenced. Two major strategies are key to improving overall outcomes. The first is a significant investment in conducting high-quality clinical trials, which is necessary to improve the evidence base for clinical dressing care. The second is to ensure that healthcare professionals using DFU dressings adhere to existing evidence-based guidance on the selection of appropriate dressings, and guidelines are needed to encourage clinicians to adopt those treatments that have been shown to be effective in robust studies, primarily in randomized controlled trials.

## Author contributions

CY and CX conceived, supervised, writing-reviewed the manuscript, cofounded and co-administrated the project. All other authors took a part in originally draft writing. Authors approved the final version.

## References

[1] McDermott K Fang M Boulton AJM Selvin E Hicks CW. Etiology, epidemiology, and disparities in the burden of diabetic foot ulcers. Diabetes Care (2023) 46(1):209–21. doi: 10.2337/dci22-0043 PMC9797649 36548709

[2] Jeon BJ Choi HJ Kang JS Tak MS Park ES. Comparison of five systems of classification of diabetic foot ulcers and predictive factors for amputation. Int Wound J (2017) 14:3. doi: 10.1111/iwj.12642 PMC7949506 27723246

[3] Sorber R Abularrage CJ. Diabetic foot ulcers: Epidemiology and the role of multidisciplinary care teams. Semin Vasc Surg (2021) 34(1):47–53. doi: 10.1053/j.semvascsurg.2021.02.006 33757635

[4] Shi C Wang C Liu H Li Q Li R Zhang Y. Selection of appropriate wound dressing for various wounds. Front Bioeng Biotechnol (2020) 8:182. doi: 10.3389/fbioe.2020.00182 32266224 PMC7096556

[5] Nuutila K Eriksson E. Moist wound healing with commonly available dressings. Adv Wound Care (2021) 10:12. doi: 10.1089/wound.2020.1232 PMC8568799 32870777

[6] Noor S Khan RU Ahmad J. Understanding diabetic foot infection and its management. Diabetes Metab Syndr (2017) 11:2. doi: 10.1016/j.dsx.2016.06.023 27377687

[7] Nkonge KM Nkonge DK Nkonge TN. Screening for diabetic peripheral neuropathy in resource-limited settings. Diabetol Metab Syndr (2023) 15(1):55. doi: 10.1186/s13098-023-01032-x 36945043 PMC10031885

[8] Volmer-Thole M Lobmann R. Neuropathy and diabetic foot syndrome. Int J Mol Sci (2016) 17:6. doi: 10.3390/ijms17060917 PMC4926450 27294922

[9] Afonso AC Oliveira D Saavedra MJ Borges A Simões M. Biofilms in diabetic foot ulcers: impact, risk factors and control strategies. Int J Mol Sci (2021) 22:15. doi: 10.3390/ijms22158278 PMC8347492 34361044

[10] Hicks CW Selvin E. Epidemiology of peripheral neuropathy and lower extremity disease in diabetes. Curr Diabetes Rep (2019) 19:10. doi: 10.1007/s11892-019-1212-8 PMC6755905 31456118

[11] Noor S Zubair M Ahmad J. Diabetic foot ulcer–A review on pathophysiology, classification and microbial etiology. Diabetes Metab Syndr (2015) 9:3. doi: 10.1016/j.dsx.2015.04.007 25982677

[12] Vouillarmet J Bourron O Gaudric J Lermusiaux P Millon A Hartemann A. Lower-extremity arterial revascularization: Is there any evidence for diabetic foot ulcer-healing? Diabetes Metab (2016) 42:1. doi: 10.1016/j.diabet.2015.05.004 26072053

[13] Lapolla A Piarulli F Sartore G Ceriello A Ragazzi E Reitano R. Advanced glycation end products and antioxidant status in type 2 diabetic patients with and without peripheral artery disease. Diabetes Care (2007) 30:3. doi: 10.2337/dc06-1508 17327339

[14] Da Silva J Leal EC Carvalho E. Bioactive antimicrobial peptides as therapeutic agents for infected diabetic foot ulcers. Biomolecules (2021) 11:12. doi: 10.3390/biom11121894 PMC8699205 34944538

[15] Darvishi S Tavakoli S Kharaziha M Girault HH Kaminski CF Mela I. Advances in the sensing and treatment of wound biofilms. Angew Chem Int Ed Engl (2022) 61:13. doi: 10.1002/anie.202112218 PMC9303468 34806284

[16] Dong J Chen L Zhang Y Jayaswal N Mezghani I Zhang W. Mast cells in diabetes and diabetic wound healing. Adv Ther (2020) 37:11. doi: 10.1007/s12325-020-01499-4 PMC7547971 32935286

[17] Theocharidis G Thomas BE Sarkar D Mumme HL Pilcher WJR Dwivedi B. Single cell transcriptomic landscape of diabetic foot ulcers. Nat Commun (2022) 13:1. doi: 10.1038/s41467-021-27801-8 35013299 PMC8748704

[18] Armstrong DG Boulton AJM Bus SA. Diabetic foot ulcers and their recurrence. N Engl J Med (2017) 376(24):2367–75. doi: 10.1056/NEJMra1615439 28614678

[19] Chen P Vilorio NC Dhatariya K Jeffcoate W Lobmann R McIntosh C. Guidelines on interventions to enhance healing of foot ulcers in people with diabetes (IWGDF 2023 update). Diabetes Metab Res Rev (2023) 25:e3644. doi: 10.1002/dmrr.3644 37232034

[20] Parizad N Hajimohammadi K Goli R. Surgical debridement, maggot therapy, negative pressure wound therapy, and silver foam dressing revive hope for patients with diabetic foot ulcer: A case report. Int J Surg Case Rep (2021) 82:105931. doi: 10.1016/j.ijscr.2021.105931 33962267 PMC8121707

[21] Moura LI Dias AM Carvalho E de Sousa HC. Recent advances on the development of wound dressings for diabetic foot ulcer treatment–a review. Acta Biomater (2013) 9:7. doi: 10.1016/j.actbio.2013.03.033 23542233

[22] Saco M Howe N Nathoo R Cherpelis B. Comparing the efficacies of alginate, foam, hydrocolloid, hydrofiber, and hydrogel dressings in the management of diabetic foot ulcers and venous leg ulcers: a systematic review and meta-analysis examining how to dress for success. Dermatol Online J (2016), 22(8):13030/qt7ph5v17z. 27617934

[23] Wang X Yuan CX Xu B Yu Z. Diabetic foot ulcers: Classification, risk factors and management. World J Diabetes (2022) 13(12):1049–65. doi: 10.4239/wjd.v13.i12.1049 PMC9791567 36578871

[24] Lazzarini PA Jarl G Gooday C Viswanathan V Caravaggi CF Armstrong DG. Effectiveness of offloading interventions to heal foot ulcers in persons with diabetes: a systematic review. Diabetes Metab Res Rev (2020) 36 Suppl 1(Suppl 1):e3275. doi: 10.1002/dmrr.3275 32176438 PMC8370012

[25] Hinchliffe RJ Forsythe RO Apelqvist J Boyko EJ Fitridge R Hong JP. Guidelines on diagnosis, prognosis, and management of peripheral artery disease in patients with foot ulcers and diabetes (IWGDF 2019 update). Diabetes Metab Res Rev (2020) 36 Suppl 1:e3276. doi: 10.1002/dmrr.3276 31958217

[26] Everett E Mathioudakis N. Update on management of diabetic foot ulcers. Ann N Y Acad Sci (2018) 1411(1):153–65. doi: 10.1111/nyas.13569 PMC5793889 29377202

[27] Xiang J Wang S He Y Xu L Zhang S Tang Z. Reasonable glycemic control would help wound healing during the treatment of diabetic foot ulcers. Diabetes Ther (2019) 10(1):95–105. doi: 10.1007/s13300-018-0536-8 30465160 PMC6349287

[28] Shi C Wang C Liu H Li Q Li R Zhang Y. Selection of appropriate wound dressing for various wounds. Front Bioeng Biotechnol (2020) 8:182. doi: 10.3389/fbioe.2020.00182 32266224 PMC7096556

[29] Simões D Miguel SP Ribeiro MP Coutinho P Mendonça AG Correia IJ. Recent advances on antimicrobial wound dressing: A review. Eur J Pharm Biopharm (2018) 127:130–141. doi: 10.1016/j.ejpb.2018.02.022 29462687

[30] Pinho E Soares G. Functionalization of cotton cellulose for improved wound healing. J Mater Chem B (2018) 6:13. doi: 10.1039/c8tb00052b 32254354

[31] Mihai MM Dima MB Dima B Holban AM. Nanomaterials for wound healing and infection control. Materials (2019) 12:13. doi: 10.3390/ma12132176 PMC6650835 31284587

[32] Dale BA Wright DH. Say goodbye to wet-to-dry wound care dressings: changing the culture of wound care management within your agency. Home Healthc Nurse (2011) 29:7. doi: 10.1097/NHH.0b013e31821b726e 21716045

[33] Cowan LJ Stechmiller J. Prevalence of wet-to-dry dressings in wound care. Adv Skin Wound Care (2009) 22(12):567–73. doi: 10.1097/01.ASW.0000363469.25740.74 19935135

[34] Ning CC Logsetty S Ghughare S Liu S. Effect of hydrogel grafting, water and surfactant wetting on the adherence of PET wound dressings. Burns (2014) 40:6. doi: 10.1016/j.burns.2013.12.024 24485358

[35] Venkatrajah B Malathy VV Elayarajah B Rajendran R Rammohan R. Synthesis of carboxymethyl chitosan and coating on wound dressing gauze for wound healing. Pak J Biol Sci (2013) 16:22. doi: 10.3923/pjbs.2013.1438.1448 24511685

[36] Fang QQ Wang XF Zhao WY Shi BH Lou D Chen CY. Development of a chitosan-vaseline gauze dressing with wound-healing properties in murine models. Am J Trop Med Hyg (2020) 102:2. doi: 10.4269/ajtmh.19-0387 PMC7008328 31802727

[37] Dong CY Liu WJ Chi RX Du H. Effect of oil gauze silver dressings on diabetic foot ulcers in the elderly. Pak J Med Scix (2017) 33:5. doi: 10.12669/pjms.335.11509 PMC5673713 29142544

[38] Wang K Chen Q Shao Y Yin S Liu C Liu Y. Anticancer activities of TCM and their active components against tumor metastasis. BioMed Pharmacother (2021) 133. doi: 10.1016/j.biopha.2020.111044 33378952

[39] Zhang Y Yuan H Kang J Xie H Long X Qi L. Clinical study for external washing by traditional Chinese medicine in the treatment of multiple infectious wounds of diabetic foot: Study protocol clinical trial (SPIRIT compliant). Medicine (2020) 99:17. doi: 10.1097/MD.0000000000019841 PMC7220697 32332634

[40] Fu Q Yang H Zhang L Liu Y Li X Dai M. Traditional Chinese medicine foot bath combined with acupoint massage for the treatment of diabetic peripheral neuropathy: A systematic review and meta-analysis of 31 RCTs. Diabetes Metab Res Rev (2020) 36:2. doi: 10.1002/dmrr.3218 31659861

[41] Li X Wang HH Xu J Tang LY Li DF Zhang Y. Study on active components of Fufang Huangbai Ye for diabetic foot treatment by UPLC-LTQ-Orbitrap-MS and network pharmacology. Zhongguo Zhong Yao Za Zhi (2019) 44:10. doi: 10.19540/j.cnki.cjcmm.20190328.201 31355569

[42] Zhong L Shi C Hou Q Yang R Li M Fu X. Promotive effects of four herbal medicine ARCC on wound healing in mice and human. Health Sci Rep (2022) 5:3. doi: 10.1002/hsr2.494 PMC9059203 35509387

[43] Zhang CS Tan HY Zhang GS Zhang AL Xue CC Xie YM. Placebo devices as effective control methods in acupuncture clinical trials: A systematic review. PloS One (2015) 10:11. doi: 10.1371/journal.pone.0140825 PMC4633221 26536619

[44] Kan Y Zhang XN Yu QQ He W Wang XY Wan HY. Moxibustion promoted transformation of inflammatory phase to facilitate wound healing in rats with full-thickness cutaneous wounds. Zhen Ci Yan Jiu (2019) 44:5. doi: 10.13702/j.1000-0607.190121 31155868

[45] Heyer K Augustin M Protz K Herberger K Spehr C Rustenbach SJ. Effectiveness of advanced versus conventional wound dressings on healing of chronic wounds: systematic review and meta-analysis. Dermatology (2013) 226(2):172–84:380. doi: 10.1159/000348331 23711429

[46] Wundauflagen HTL. Übersicht und Klassifikation [Wound dressings. Overview and classification]. Unfallchirurg (2012) 115(9):774–82. doi: 10.1007/s00113-012-2209-9 22935895

[47] British Medical Association and Royal Pharmaceutical Society of Great Britain. British National Formulary Appendix 8: Wound management products and elastic hosiery (2014). Available at: http://www.bnf.org.uk/bnf/bnf/current (Accessed November 2014).

[48] Wu L Norman G Dumville JC O’Meara S Bell-Syer SE. Dressings for treating foot ulcers in people with diabetes: an overview of systematic reviews. Cochrane Database Syst Rev (2015) 7. doi: 10.1002/14651858.CD010471.pub2 PMC7083265 26171906

[49] Liang Y He J Guo B. Functional hydrogels as wound dressing to enhance wound healing. ACS Nano (2021) 15:8. doi: 10.1021/acsnano.1c04206 34374515

[50] Miguel SP Ribeiro MP Brancal H Coutinho P Correia IJ. Thermoresponsive chitosan-agarose hydrogel for skin regeneration. Carbohydr Polym (2014) 111:366–73. doi: 10.1016/j.carbpol.2014.04.093 25037363

[51] Zenda S Ishi S Kawashima M Arahira S Tahara M Hayashi R. A Dermatitis Control Program (DeCoP) for head and neck cancer patients receiving radiotherapy: a prospective phase II study. Int J Clin Oncol (2013) 18(2):350–5. doi: 10.1007/s10147-012-0385-9 22350025

[52] Pal K Banthia AK Majumdar DK. Polymeric Hydrogels: Characterization and Biomedical Applications. Monomers polym (2009) 12:197–220. doi: 10.1163/156855509X436030

[53] Yang M Ding L Liu R. Application effect of comprehensive nursing intervention in patients with diabetic foot. Panminerva Med (2021). doi: 10.23736/S0031-0808.20.04257-3 33496156

[54] Zhou L Lei D Wang Q Luo X Chen Y. Biocompatible Polyphosphorylcholine Hydrogels with Inherent Antibacterial and Nonfouling Behavior Effectively Promote Skin Wound Healing. ACS Appl Bio Mater (2020) 3(8):5357–5366. doi: 10.1021/acsabm.0c00666 35021710

[55] Moura LI Dias AM Carvalho E de Sousa HC. Recent advances on the development of wound dressings for diabetic foot ulcer treatment–a review. Acta biomater (2013) 9(7):7093–114. doi: 10.1016/j.actbio.2013.03.033 23542233

[56] Zhang L Liu M Zhang Y Pei R. Recent Progress of Highly Adhesive Hydrogels as Wound Dressings. Biomacromolecules (2020) 21(10):3966–3983. doi: 10.1021/acs.biomac.0c01069 32960043

[57] Wang H Xu Z Zhao M Liu G Wu J. Advances of hydrogel dressings in diabetic wounds. Biomater Sci (2021) 9:5. doi: 10.1039/d0bm01747g 33433534

[58] Alven S Peter S Mbese Z Aderibigbe BA. Polymer-based wound dressing materials loaded with bioactive agents: potential materials for the treatment of diabetic wounds. Polymers (2022) 14:4. doi: 10.3390/polym14040724 PMC8874614 35215637

[59] Caló E Khutoryanskiy VV Eur. Polym. J. (2015) 65:252–67.

[60] Xian C Yuan Q Bao Z Liu G Wu J. Progress on intelligent hydrogels based on RAFT polymerization: design strategy, fabrication and the applications for controlled drug delivery. Chin Chem Lett (2020) 31(01):19–27. doi: 10.1016/j.cclet.2019.03.052

[61] Wu H Ni R Shi Y Hu Y Shen Z Pang Q. The promising hydrogel candidates for preclinically treating diabetic foot ulcer: A systematic review and meta-analysis. Adv Wound Care (New Rochelle) (2023) 12(1):28–37. doi: 10.1089/wound.2021.0162 35229628

[62] Frykberg RG Banks J. Challenges in the Treatment of Chronic Wounds. Adv Wound Care (2015) 4(9):560–82. doi: 10.1089/wound.2015.0635 PMC4528992 26339534

[63] Phillipson M Kubes P. The Healing Power of Neutrophils. Trends Immunol (2019) 40(7):635–47. doi: 10.1016/j.it.2019.05.001 31160208

[64] Chen H Chang X Du D Li J Xu H Yang X. Microemulsion-based hydrogel formulation of ibuprofen for topical delivery. Int J Pharm (2006) 315(1-2):52–8. doi: 10.1016/j.ijpharm.2006.02.015 16600540

[65] Thakur VK Thakur MKJ. Nanocellulose-Based Polymer Nanocomposites: An Introduction. Cleaner Prod (2014), 1–15. doi: 10.1002/9781118872246.ch1

[66] Hou C Chen L Yang L Ji X. An insight into anti-inflammatory effects of natural polysaccharides. Int J Biol Macromol (2020) 153:248–55. doi: 10.1016/j.ijbiomac.2020.02.315 32114173

[67] Wathoni N Motoyama K Higashi T Okajima M Kaneko T Arima H. Physically crosslinked-sacran hydrogel films for wound dressing application. Int J Biol Macromol (2016) 89:465–70. doi: 10.1016/j.ijbiomac.2016.05.006 27151668

[68] Sudhadevi T Vijayakumar HS Hariharan EV Sandhyamani S Krishnan LK. Optimizing fibrin hydrogel toward effective neural progenitor cell delivery in spinal cord injury. BioMed Mater (2021) 17:1. doi: 10.1088/1748-605X/ac3680 34736245

[69] Gong C Wu Q Wang Y Zhang D Luo F Zhao X. A biodegradable hydrogel system containing curcumin encapsulated in micelles for cutaneous wound healing. Biomaterials (2013) 34:27. doi: 10.1016/j.biomaterials.2013.05.005 23726229

[70] Augustine R Hasan A Dalvi YB Rehman SRU Varghese R Unni RN. Growth factor loaded in *situ* photocrosslinkable poly(3-hydroxybutyrate-co-3-hydroxyvalerate)/gelatin methacryloyl hybrid patch for diabetic wound healing. Mater Sci Eng C Mater Biol Appl (2021) 118. doi: 10.1016/j.ms.2020.111519 33255074

[71] Rustad KC Wong VW Sorkin M Glotzbach JP Major MR Rajadas J. Enhancement of mesenchymal stem cell angiogenic capacity and stemness by a biomimetic hydrogel scaffold. Biomaterials (2012) 33:1. doi: 10.1016/j.biomaterials.2011.09.041 21963148 PMC3997302

[72] Chen J Chen D Chen J Shen T Jin T Zeng B. An all-in-one CO gas therapy-based hydrogel dressing with sustained insulin release, anti-oxidative stress, antibacterial, and anti-inflammatory capabilities for infected diabetic wounds. Acta Biomater (2022) 146:49–65. doi: 10.1016/j.actbio.2022.04.043 35500813

[73] Chijcheapaza-Flores H Tabary N Chai F Maton M Staelens JN Cazaux F. Injectable chitosan-based hydrogels for trans-cinnamaldehyde delivery in the treatment of diabetic foot ulcer infections. Gels (2023) 9(3):262. doi: 10.3390/gels9030262 36975711 PMC10048173

[74] Bolton L. Diabetic foot ulcer: treatment challenges. Wounds (2022) 34(6):175–7. doi: 10.25270/wnds/2022.175177 35881427

[75] Arampatzis AS Kontogiannopoulos KN Theodoridis K Aggelidou E WillemsA RA Tsivintzelis I. Electrospun wound dressings containing bioactive natural products: physico-chemical characterization and biological assessment. Biomater Res (2021) 25:1. doi: 10.1186/s40824-021-00223-9 34271983 PMC8284004

[76] Punjataewakupt A Napavichayanun S Aramwit P. The downside of antimicrobial agents for wound healing. Eur J Clin Microbiol Infect Dis (2018) 38:1. doi: 10.1007/s10096-018-3393-5 30291466

[77] Whiting DR Guariguata L Weil C Shaw J. IDF diabetes atlas: global estimates of the prevalence of diabetes for 2011 and 2030. Diabetes Res Clin Pract (2011) 94:3. doi: 10.1016/j.diabres.2011.10.029 22079683

[78] Westphal C Neame IM Harrison JC Bower VM Gurr JM. A diabetic foot ulcer pilot study: does silicone gel sheeting reduce the incidence of reulceration? J Am Podiatr Med Assoc (2011) 101:2. doi: 10.7547/1010116 21406694

[79] Dumville JC Deshpande S O’Meara S Speak K. Hydrocolloid dressings for healing diabetic foot ulcers. Cochrane Database Syst Rev (2013) 2013:8. doi: 10.1002/14651858.CD009099.pub3 PMC7111300 23922167

[80] Duckworth PF Maddocks SE Rahatekar SS Barbour ME. Alginate films augmented with chlorhexidine hexametaphosphate particles provide sustained antimicrobial properties for application in wound care. J Mater Sci Mater Med (2020) 31:3. doi: 10.1007/s10856-020-06370-0 PMC7066275 32162052

[81] Broussard KC Powers JG. Wound dressings: selecting the most appropriate type. Am J Clin Dermatol (2013) 14:6. doi: 10.1007/s40257-013-0046-4 24062083

[82] He X Ding Y Xie W Sun R Hunt NC Song J. Rubidium-containing calcium alginate hydrogel for antibacterial and diabetic skin wound healing applications. ACS Biomater Sci Eng (2019) 7(6):2858. doi: 10.1021/acsbiomaterials.9b00547 33988359

[83] Donaghue VM Chrzan JS Rosenblum BI Giurini JM Habershaw GM Veves A. Evaluation of a collagen-alginate wound dressing in the management of diabetic foot ulcers. Adv Wound Care (1998) 11:3. doi: 10.25270/wnds/2021.169177 9729942

[84] Motzkau M Tautenhahn J Lehnert H Lobmann R. Expression of matrix-metalloproteases in the fluid of chronic diabetic foot wounds treated with a protease absorbent dressing. Exp Clin Endocrinol Diabetes (2011) 119:5. doi: 10.1055/s-0030-1267235 21031342

[85] Kim BY Kim CH Jung CH Mok JO Suh KI Kang SK. Association between subclinical hypothyroidism and severe diabetic retinopathy in Korean patients with type 2 diabetes. Endocr J (2011) 58:12. doi: 10.1507/endocrj.ej11-0199 21931224

[86] Tanaka R Inoue H Ishikawa T Ichikawa Y Sato R Shimizu A. Use of sponge-foam inserts in compression bandaging of non-healing venous leg ulcers. Ann Vasc Dis (2021) 14:1. doi: 10.3400/avd.oa.20-00159 33786099 PMC7991709

[87] Carter MJ Tingley-Kelley K Warriner RA 3rd. Silver treatments and silver-impregnated dressings for the healing of leg wounds and ulcers: a systematic review and meta-analysis. J Am Acad Dermatol (2010) 63:4. doi: 10.1016/j.jaad.2009.09.007 20471135

[88] Bergin SM Wraight P. Silver based wound dressings and topical agents for treating diabetic foot ulcers. Cochrane Database Syst Rev (2006) 25(1):CD005082. doi: 10.1002/14651858.CD005082.pub2 16437516

[89] Wang YC Lee HC Chen CL Kuo MC Ramachandran S Chen RF. The effects of silver-releasing foam dressings on diabetic foot ulcer healing. J Clin Med (2021) 10:7. doi: 10.3390/jcm10071495 PMC8038333 33916790

[90] Mobed-Miremadi M Nagendra RK Ramachandruni SL Rook JJ Keralapura M Goedert M. Polystyrene microsphere and 5-fluorouracil release from custom-designed wound dressing films. Prog Biomater (2013) 2:1. doi: 10.1186/2194-0517-2-1 29470744 PMC5151105

[91] Burkatovskaya M Tegos GP Swietlik E Demidova TN P Castano A Hamblin MR. Use of chitosan bandage to prevent fatal infections developing from highly contaminated wounds in mice. Biomaterials (2006) 27:22. doi: 10.1016/j.biomaterials.2006.03.028 PMC2935802 16616364

[92] Wang JX Si HJ Yu SL Wu YX Wu YF Wang Y. Analysis of distribution characteristics and risk factors of multidrug-resistant bacteria infection in diabetic foot patients. Clin J Med Offic (2022) 50:2. doi: 10.1186/s12902-022-00957-0

[93] Guiomar AJ Urbano AM. Polyhexanide-releasing membranes for antimicrobial wound dressings: A critical review. Membranes (Basel) (2022) 12(12):1281. doi: 10.3390/membranes12121281 36557188 PMC9781366

[94] Liang Y Liang Y Zhang H Guo B. Antibacterial biomaterials for skin wound dressing. Asian J Pharm Sci (2022) 17:3. doi: 10.1016/j.ajps.2022.01.001 PMC9237601 35782328

[95] Deng SH Lurant DSL Cheng QF Liu ZP. Efficacy and safety of honey dressing in the treatment of diabetic foot ulcers: a Meta analysis. Chin J Diabetes Mellitus (2019) 11:7. doi: 10.3760/cma.j.issn.1674-5809.2019.07.008

[96] Martinotti S Ranzato E. Honey, wound repair and regenerative medicine. J Funct Biomater (2018) 9:2. doi: 10.3390/jfb9020034 PMC6023338 29738478

[97] Yuan L Li X. Study on the method of local external application of honey in the treatment of diabetic foot. Chin Community Doctors (2018) 34:7. doi: 10.3969/j.issn.1007-614x.2018.7.77

[98] Dayya D O’Neill OJ Huedo-Medina TB Habib N Moore J Iyer K. Debridement of diabetic foot ulcers. Adv Wound Care (New Rochelle) (2022) 11:12. doi: 10.1089/wound.2021.0016 PMC9527061 34376065

[99] Chen HH Chen L Qi AQ Yu JQ. Clinical observation of NPWT combined with silver ion hydrogel dressing in the treatment of diabetic foot ulcer. J Bengbu Med Coll (2019) 44:9. doi: 10.13898/j.cnki.issn.1000-2200.2019.09.019

[100] Jung WK Koo HC Kim KW Shin S Kim SH Park YH. Antibacterial activity and mechanism of action of the silver ion in Staphylococcus aureus and Escherichia coli. Appl Environ Microbiol (2008) 74:7. doi: 10.1128/AEM.02001-07 PMC2292600 18245232

[101] Verma J Kanoujia J Parashar P Tripathi CB Saraf SA. Wound healing applications of sericin/chitosan-capped silver nanoparticles incorporated hydrogel. Drug Deliv Transl Res (2017) 7:1. doi: 10.1007/s13346-016-0322-y 27565984

[102] Wang M Wang C Chen M Xi Y Cheng W Mao C. Efficient angiogenesis-based diabetic wound healing/skin reconstruction through bioactive antibacterial adhesive ultraviolet shielding nanodressing with exosome release. ACS Nano (2019) 13:9. doi: 10.1021/acsnano.9b03656 31483606

[103] Li J Zhai D Lv F Yu Q Ma H Yin J. Preparation of copper-containing bioactive glass/eggshell membrane nanocomposites for improving angiogenesis, antibacterial activity and wound healing. Acta Biomater (2016) 36:254–66. doi: 10.1016/j.actbio.2016.03.011 26965395

[104] Mao C Xian Y Liu X Cui Z Yang X Yeung KWK. Photo-inspired antibacterial activity and wound healing acceleration by hydrogel embedded with ag/ag@AgCl/znO nanostructures. ACS Nano (2017) 11:9. doi: 10.1021/acsnano.7b03513 28825807

[105] Li Y Xu T Tu Z Dai W Xue Y Tang C. Bioactive antibacterial silica-based nanocomposites hydrogel scaffolds with high angiogenesis for promoting diabetic wound healing and skin repair. Theranostics (2020) 12(10):4599–600: doi: 10.7150/thno.41839 PMC7163448 32308759

[106] Gaspar-Pintiliescu A Stanciuc AM Craciunescu O. Natural composite dressings based on collagen, gelatin and plant bioactive compounds for wound healing: A review. Int J Biol Macromol (2019) 138:854–65. doi: 10.1016/j.ijbiomac.2019.07.155 31351963

[107] Argyropoulos AJ Robichaud P Balimunkwe RM Fisher GJ Hammerberg C Yan Y. Alterations of dermal connective tissue collagen in diabetes: molecular basis of aged-appearing skin. PloS One (2016) 11:4. doi: 10.1371/journal.pone.0153806 PMC4841569 27104752

[108] Lazurko C Khatoon Z Goel K Sedlakova V Eren Cimenci C Ahumada M. Multifunctional nano and collagen-based therapeutic materials for skin repair. ACS Biomater Sci Eng (2020) 6:2. doi: 10.1021/acsbiomaterials.9b01281 33464871

[109] Edwards N Feliers D Zhao Q Stone R Christy R Cheng X. An electrochemically deposited collagen wound matrix combined with adipose-derived stem cells improves cutaneous wound healing in a mouse model of type 2 diabetes. J Biomater Appl (2018) 33:4. doi: 10.1177/0885328218803754 30326802

[110] Ramanathan G Singaravelu S Muthukumar T Thyagarajan S Perumal PT Sivagnanam UT. Design and characterization of 3D hybrid collagen matrixes as a dermal substitute in skin tissue engineering. Mater Sci Eng C Mater Biol Appl (2017) 72:359–370. doi: 10.1016/j.ms.2016.11.095 28024598

[111] McGrath M Zimkowska K KJ G Maughan J Gutierrez Gonzalez J Browne S. A biomimetic, bilayered antimicrobial collagen-based scaffold for enhanced healing of complex wound conditions. ACS Appl Mater Interfaces (2023) 15(14):17444–58. doi: 10.1021/acsami.2c18837 PMC10103052 37001059

[112] Naomi R Fauzi MB. Cellulose/collagen dressings for diabetic foot ulcer: A review. Pharmaceutics (2020) 12(9):881. doi: 10.3390/pharmaceutics12090881 32957476 PMC7558961

[113] Abd El-Hack ME El-Saadony MT Shafi ME Zabermawi NM Arif M Batiha GE. Antimicrobial and antioxidant properties of chitosan and its derivatives and their applications: A review. Int J Biol Macromol (2020) 164:2726–2744. doi: 10.1016/j.ijbiomac.2020.08.153 32841671

[114] Wegrzynowska-Drzymalska K Mlynarczyk DT Chelminiak-Dudkiewicz D Kaczmarek H Goslinski T Ziegler-Borowska M. Chitosan-gelatin films cross-linked with dialdehyde cellulose nanocrystals as potential materials for wound dressings. Int J Mol Sci (2022) 23:17. doi: 10.3390/ijms23179700 PMC9456065 36077096

[115] Liu H Wan C Li C Qin Y Wang Z Yang F. A functional chitosan-based hydrogel as a wound dressing and drug delivery system in the treatment of wound healing. RSC Adv (2018) 8:14. doi: 10.1039/c7ra13510f PMC9078458 35539132

[116] Dong F Li S. Wound dressings based on chitosan-dialdehyde cellulose nanocrystals-silver nanoparticles: mechanical strength, antibacterial activity and cytotoxicity. Polymers (Basel) (2018) 10:6. doi: 10.3390/polym10060673 PMC6404142 30966707

[117] Jayaramudu T Varaprasad K Pyarasani RD Reddy KK Kumar KD Akbari-Fakhrabadi A. Chitosan capped copper oxide/copper nanoparticles encapsulated microbial resistant nanocomposite films. Int J Biol Macromol (2019) 128:499–508. doi: 10.1016/j.ijbiomac.2019.01.145 30699337

[118] Yin M Wan S Ren X Chu CC. Development of inherently antibacterial, biodegradable, and biologically active chitosan/pseudo-protein hybrid hydrogels as biofunctional wound dressings. ACS Appl Mater Interfaces (2021) 13:12. doi: 10.1021/acsami.0c21680 33739108

[119] Zhang Y Dang Q Liu C Yan J Cha D Liang S. Synthesis, characterization, and evaluation of poly(aminoethyl) modified chitosan and its hydrogel used as antibacterial wound dressing. Int J Biol Macromol (2017) 102:457–467. doi: 10.1016/j.ijbiomac.2017.04.049 28416398

[120] Li C Jiang T Zhou C Jiang A Lu C Yang G. Injectable self-healing chitosan-based POSS-PEG hybrid hydrogel as wound dressing to promote diabetic wound healing. Carbohydr Polym (2023) 299:120198. doi: 10.1016/j.carbpol.2022.120198 36876768

[121] Yu Y Tian R Zhao Y Qin X Hu L JJ Z. Self-assembled corrole/chitosan photothermal nanoparticles for accelerating infected diabetic wound healing. Adv Healthc Mater (2023) 12(16):e2201651. doi: 10.1002/adhm.202201651 36168853

[122] Hosseinzadeh A Zamani A Johari HG Vaez A Golchin A Tayebi L. Moving beyond nanotechnology to uncover a glimmer of hope in diabetes medicine: Effective nanoparticle-based therapeutic strategies for the management and treatment of diabetic foot ulcers. Cell Biochem Funct (2023) 41(5):517–41. doi: 10.1002/cbf.3816 37282756

[123] Ashwini T Prabhu A Baliga V Bhat S Thenkondar ST Nayak Y. Transforming wound management: nanomaterials and their clinical impact. Pharmaceutics (2023) 15(5):1560. doi: 10.3390/pharmaceutics15051560 37242802 PMC10221108

[124] Lin H BoLatai A Wu N. Application progress of nano silver dressing in the treatment of diabetic foot. Diabetes Metab Syndr Obes (2021) 14:4145–4154. doi: 10.2147/DMSO.S330322 PMC8491782 34621128

[125] Singh K Yadav VB Yadav U Nath G Srivastava A Zamboni P. Evaluation of biogenic nanosilver-acticoat for wound healing: A tri-modal in silico, in *vitro* and in *vivo* study, Colloids and Surfaces A. Physicochemical Eng Aspects (2023) 670:131575. doi: 10.1016/j.colsurfa.2023.131575

[126] Zhang K Li Y He J Xu J Wan Y Wan S. Therapeutic effect of epidermal growth factor combined with nano silver dressing on diabetic foot patients. Front Pharmacol (2021) 12:627098. doi: 10.3389/fphar.2021.627098 33967761 PMC8102863

[127] Lou P Liu S Wang Y Pan C Xu X Zhao M. Injectable self-assembling peptide nanofiber hydrogel as a bioactive 3D platform to promote chronic wound tissue regeneration. Acta Biomater (2021) 135:100–112. doi: 10.1016/j.actbio.2021.08.008 34389483

[128] Chang T Yin H Yu X Wang L Fan L Xin JH. 3D PCL/collagen nanofibrous medical dressing for one-time treatment of diabetic foot ulcers. Colloids Surf B Biointerfaces (2022) 214. doi: 10.1016/j.colsurfb.2022.112480 35358884

[129] Li G Liu H Yi J Pu F Ren J Qu X. Integrating incompatible nanozyme-catalyzed reactions for diabetic wound healing. Small (2022) 19(10):e2206707. doi: 10.1002/smll.202206707 36541749

[130] Hassiba AJ El Zowalaty ME Webster TJ Abdullah AM Nasrallah GK Khalil KA. Synthesis, characterization, and antimicrobial properties of novel double layer nanocomposite electrospun fibers for wound dressing applications. Int J Nanomed (2017) 12:2205–2213. doi: 10.2147/IJN.S123417 PMC5367563 28356737

[131] Li X Wang C Yang S Liu P Zhang B. Electrospun PCL/mupirocin and chitosan/lidocaine hydrochloride multifunctional double layer nanofibrous scaffolds for wound dressing applications. Int J Nanomed (2018) 13:5287–5299. doi: 10.2147/IJN.S177256 PMC6136417 30237715

[132] Pedram R Mokhtari J Abbasi M. Calendula officinalis extract/PCL/Zein/Gum arabic nanofibrous bio-composite scaffolds via suspension, two-nozzle and multilayer electrospinning for skin tissue engineering. Int J Biol Macromol (2019) 135:530–543. doi: 10.1016/j.ijbiomac.2019.05.204 31152839

[133] El-Aassar MR El-Beheri NG Agwa MM Eltaher HM Alseqely M Sadik WS El-Khordagui L. Antibiotic-free combinational hyaluronic acid blend nanofibers for wound healing enhancement. Int J Biol Macromol (2021) 167:1552–1563. doi: 10.1016/j.ijbiomac.2020.11.109 33212109

[134] Liu L Cai R Wang Y Tao G Ai L Wang P. Polydopamine-assisted silver nanoparticle self-assembly on sericin/agar film for potential wound dressing application. Int J Mol Sci (2018) 19:10. doi: 10.3390/ijms19102875 PMC6213261 30248951

[135] Cai R Tao G He H Song K Zuo H Jiang W. One-step synthesis of silver nanoparticles on polydopamine-coated sericin/polyvinyl alcohol composite films for potential antimicrobial applications. Molecules (2017) 22:5. doi: 10.3390/molecules22050721 PMC6154384 28468293

[136] He H Cai R Wang Y Tao G Guo P Zuo H. Preparation and characterization of silk sericin/PVA blend film with silver nanoparticles for potential antimicrobial application. Int J Biol Macromol (2017) 104:457–464. doi: 10.1016/j.ijbiomac.2017.06.009 28619637

[137] Muñoz-Escobar A Ruíz-Baltazar ÁJ Reyes-López SY. Novel route of synthesis of PCL-cuONPs composites with antimicrobial properties. Dose Response (2019) 17:3. doi: 10.1177/1559325819869502 PMC6699009 31452651

[138] Zhu G Sun Z Hui P Chen W Jiang X. Composite film with antibacterial gold nanoparticles and silk fibroin for treating multidrug-resistant E. coli-infected wounds. ACS Biomater Sci Eng (2021) 7:5. doi: 10.1021/acsbiomaterials.0c01271 33966376

[139] Li Y Tian Y Zheng W Feng Y Huang R Shao J. Composites of bacterial cellulose and small molecule-decorated gold nanoparticles for treating gram-negative bacteria-infected wounds. Small (2017) 13:27. doi: 10.1002/smll.201700130 28544761

[140] Ai L Wang Y Tao G Zhao P Umar A Wang P. Polydopamine-based surface modification of znO nanoparticles on sericin/polyvinyl alcohol composite film for antibacterial application. Molecules (2019) 24:3. doi: 10.3390/molecules24030503 PMC6384743 30704137

[141] Ahmed R Tariq M Ali I Asghar R Noorunnisa Khanam P Augustine R. Novel electrospun chitosan/polyvinyl alcohol/zinc oxide nanofibrous mats with antibacterial and antioxidant properties for diabetic wound healing. Int J Biol Macromol (2018) 120. doi: 10.1016/j.ijbiomac.2018.08.057 30110603

[142] Ezhilarasu H RaMalingam R Dhand C Lakshminarayanan R Sadiq A Gandhimathi C. Biocompatible aloe vera and tetracycline hydrochloride loaded hybrid nanofibrous scaffolds for skin tissue engineering. Int J Mol Sci (2019) 20:20. doi: 10.3390/ijms20205174 PMC6834217 31635374

[143] Baghersad S Hajir Bahrami S Mohammadi MR Mojtahedi MRM Milan PB. Development of biodegradable electrospun gelatin/aloe-vera/poly(ϵ-caprolactone) hybrid nanofibrous scaffold for application as skin substitutes. Mater Sci Eng C Mater Biol Appl (2018) 93:367–379. doi: 10.1016/j.msec.2018.08.020 30274069

[144] Eskandarinia A Kefayat A Agheb M Rafienia M Amini Baghbadorani M Navid S. A novel bilayer wound dressing composed of a dense polyurethane/propolis membrane and a biodegradable polycaprolactone/gelatin nanofibrous scaffold. Sci Rep (2020) 10:1. doi: 10.1038/s41598-020-59931-2 32080256 PMC7033255

[145] Eskandarinia A Kefayat A Gharakhloo M Agheb M Khodabakhshi D Khorshidi M. A propolis enriched polyurethane-hyaluronic acid nanofibrous wound dressing with remarkable antibacterial and wound healing activities. Int J Biol Macromol (2020) 149:467–476. doi: 10.1016/j.ijbiomac.2020.01.255 32001284

[146] Hussein MAM Gunduz O Sahin A Grinholc M El-Sherbiny IM Megahed M. Dual spinneret electrospun polyurethane/PVA-gelatin nanofibrous scaffolds containing cinnamon essential oil and nanoceria for chronic diabetic wound healing: preparation, physicochemical characterization and *in-vitro* evaluation. Molecules (2022) 27:7. doi: 10.3390/molecules27072146 PMC9000402 35408546

[147] Ardekani NT Khorram M Zomorodian K Yazdanpanah S Veisi H Veisi H. Evaluation of electrospun poly (vinyl alcohol)-based nanofiber mats incorporated with Zataria multiflora essential oil as potential wound dressing. Int J Biol Macromol (2019) 125:743–750. doi: 10.1016/j.ijbiomac.2018.12.085 30543881

[148] Dong X Wu W Pan P Zhang XZ. Engineered living materials for advanced diseases therapy. Adv Mater (2023):e2304963. doi: 10.1002/adma.202304963 37436776

[149] Sethuram L Thomas J Mukherjee A Chandrasekaran N. A review on contemporary nanomaterial-based therapeutics for the treatment of diabetic foot ulcers (DFUs) with special reference to the Indian scenario. Nanoscale Adv (2022) 4(11):2367–98. doi: 10.1039/d1na00859e PMC9418054 36134136

[150] O’Loughlin A Kulkarni M Vaughan EE Creane M Liew A Dockery P. Autologous circulating angiogenic cells treated with osteopontin and delivered via a collagen scaffold enhance wound healing in the alloxan-induced diabetic rabbit ear ulcer model. Stem Cell Res Ther (2013) 4:6. doi: 10.1186/scrt388 24444259 PMC4054999

[151] Bui L Edwards S Hall E Alderfer L Round K Owen M. Engineering bioactive nanoparticles to rejuvenate vascular progenitor cells. Commun Biol (2022) 5:1. doi: 10.1038/s42003-022-03578-4 35768543 PMC9243106

[152] Silva EA Kim ES Kong HJ Mooney DJ. Material-based deployment enhances efficacy of endothelial progenitor cells. Proc Natl Acad Sci USA (2008) 105:38. doi: 10.1073/pnas.0803873105 PMC2567164 18794520

[153] Lopes L Setia O Aurshina A Liu S Hu H Isaji T. Stem cell therapy for diabetic foot ulcers: a review of preclinical and clinical research. Stem Cell Res Ther (2018) 9(1):188. doi: 10.1186/s13287-018-0938-6 29996912 PMC6042254

[154] Verdi J Shirian S Saleh M Khadem Haghighian H Kavianpour M. Mesenchymal stem cells regenerate diabetic foot ulcers: A review article. World J Plast Surg (2022) 11(1):12–22. doi: 10.52547/wjps.11.1.12 35592239 PMC9018029

[155] Cao Y Gang X Sun C Wang G. Mesenchymal stem cells improve healing of diabetic foot ulcer. J Diabetes Res (2017) 2017:9328347. doi: 10.1155/2017/9328347 28386568 PMC5366201

[156] Park SR Kim JW Jun HS Roh JY Lee HY Hong IS. Stem cell secretome and its effect on cellular mechanisms relevant to wound healing. Mol Ther (2018) 26(2):606–17. doi: 10.1016/j.ymthe.2017.09.023 PMC5835016 29066165

[157] O’Loughlin A Kulkarni M Creane M Vaughan EE Mooney E Shaw G. Topical administration of allogeneic mesenchymal stromal cells seeded in a collagen scaffold augments wound healing and increases angiogenesis in the diabetic rabbit ulcer. Diabetes (2013) 62:7. doi: 10.2337/db12-1822 PMC3712062 23423568

[158] Tong C Hao H Xia L Liu J Ti D Dong L. Hypoxia pretreatment of bone marrow-derived mesenchymal stem cells seeded in a collagen-chitosan sponge scaffold promotes skin wound healing in diabetic rats with hindlimb ischemia. Wound Repair Regener (2016) 24:1. doi: 10.1111/wrr.12369 26463737

[159] Guo J Hu H Gorecka J Bai H He H Assi R. Adipose-derived mesenchymal stem cells accelerate diabetic wound healing in a similar fashion as bone marrow-derived cells. Am J Physiol Cell Physiol (2018) 315:6. doi: 10.1152/ajpcell.00120.2018 PMC6336941 30404559

[160] Wu YY Jiao YP Xiao LL Li MM Liu HW Li SH. Experimental study on effects of adipose-derived stem cell-seeded silk fibroin chitosan film on wound healing of a diabetic rat model. Ann Plast Surg (2018) 80:5. doi: 10.1097/SAP.0000000000001355 29443833 PMC5916459

[161] da Silva LP Santos TC Rodrigues DB Pirraco RP Cerqueira MT Reis RL. Stem cell-containing hyaluronic acid-based spongy hydrogels for integrated diabetic wound healing. J Invest Dermatol (2017) 137:7. doi: 10.1016/j.jid.2017.02.976 28259681

[162] Assi R Foster TR He H Stamati K Bai H Huang Y. Delivery of mesenchymal stem cells in biomimetic engineered scaffolds promotes healing of diabetic ulcers. Regener Med (2016) 11:3. doi: 10.2217/rme-2015-0045 PMC4976993 26986810

[163] Bai H Kyu-Cheol N Wang Z Cui Y Liu H Liu H. Regulation of inflammatory microenvironment using a self-healing hydrogel loaded with BM-MSCs for advanced wound healing in rat diabetic foot ulcers. J Tissue Eng (2020) 11. doi: 10.1177/2041731420947242 PMC7444096 32913623

[164] Dixon D Edmonds M. Managing diabetic foot ulcers: pharmacotherapy for wound healing. Drugs (2021) 81(1):29–56. doi: 10.1007/s40265-020-01415-8 33382445

[165] Ardizzone A Bova V Casili G Repici A Lanza M Giuffrida R. Role of basic fibroblast growth factor in cancer: biological activity, targeted therapies, and prognostic value. Cells (2023) 12(7):1002. doi: 10.3390/cells12071002 37048074 PMC10093572

[166] Peng J Zhao H Tu C Xu Z Ye L Zhao L. *In situ* hydrogel dressing loaded with heparin and basic fibroblast growth factor for accelerating wound healing in rat. Mater Sci Eng C Mater Biol Appl (2020) 116:111169. doi: 10.1016/j.ms.2020.111169 32806292

[167] Ding R Zhu S Zhao X Yue R. Vascular endothelial growth factor levels in diabetic peripheral neuropathy: a systematic review and meta-analysis. Front Endocrinol (Lausanne) (2023) 14:1169405. doi: 10.3389/fendo.2023.1169405 37251664 PMC10213658

[168] Li G Zou X Zhu Y Zhang J Zhou L Wang D. Expression and influence of matrix metalloproteinase-9/tissue inhibitor of metalloproteinase-1 and vascular endothelial growth factor in diabetic foot ulcers. Int J Low Extrem Wounds (2017) 16(1):6–13. doi: 10.1177/1534734617696728 28682675

[169] Batrakova EV Kim MS. Using exosomes, naturally-equipped nanocarriers, for drug delivery. J Control Release (2015) 219:396–405. doi: 10.1016/j.jconrel.2015.07.030 26241750 PMC4656109

[170] Liang ZH NF P SS L ZY Q Liang P Wang J. Exosomes from mmu_circ_0001052-modified adipose-derived stem cells promote angiogenesis of DFU via miR-106a-5p and FGF4/p38MAPK pathway. Stem Cell Res Ther (2022) 13(1):336. doi: 10.1186/s13287-022-03015-7 35870977 PMC9308214

[171] Chen C Wang Q Li D Qi Z Chen Y Wang S. MALAT1 participates in the role of platelet-rich plasma exosomes in promoting wound healing of diabetic foot ulcer. Int J Biol Macromol (2023) 238:124170. doi: 10.1016/j.ijbiomac.2023.124170 36963542

[172] Tian Y Zhang T Li J Tao Y. Advances in development of exosomes for ophthalmic therapeutics. Adv Drug Deliv Rev (2023) 199:114899. doi: 10.1016/j.addr.2023.114899 37236425

[173] Orban YA Soliman MA Hegab YH Alkilany MM. Autologous platelet-rich plasma vs conventional dressing in the management of chronic diabetic foot ulcers. Wounds (2022) 33:2. doi: 10.25270/wnds/2022.3642 35108667

[174] Elsaid A El-Said M Emile S Youssef M Khafagy W Elshobaky A. Randomized controlled trial on autologous platelet-rich plasma versus saline dressing in treatment of non-healing diabetic foot ulcers. World J Surg (2020) 44:4. doi: 10.1007/s00268-019-05316-0 31811339

[175] Ahmed M Reffat SA Hassan A Eskander F. Platelet-rich plasma for the treatment of clean diabetic foot ulcers. Ann Vasc Surg (2017) 38:206–211. doi: 10.1016/j.avsg.2016.04.023 27522981

[176] Boswell SG Cole BJ Sundman EA Karas V Fortier LA. Platelet-rich plasma: a milieu of bioactive factors. Arthroscopy (2012) 28(3):429–39. doi: 10.1016/j.arthro.2011.10.018 22284405

[177] Andia I Maffulli N. Platelet-rich plasma for managing pain and inflammation in osteoarthritis. Nat Rev Rheumatol (2013) 9(12):721–30. doi: 10.1038/nrrheum.2013.141 24080861

[178] Troha K Vozel D Arko M Bedina Zavec A Dolinar D Hočevar M. Autologous platelet and extracellular vesicle-rich plasma as therapeutic fluid: A review. Int J Mol Sci (2023) 24(4):3420. doi: 10.3390/ijms24043420 36834843 PMC9959846

[179] Qin X Wang J. Clinical study of local injection of autologous platelet-rich plasma in treatment of diabetic foot ulcer. Zhongguo Xiu Fu Chong Jian Wai Ke Za Zhi (2019) 33:12. doi: 10.7507/1002-1892.201905124 PMC8355805 31823556

[180] He Z Liu A Yu J Chen X. Role of platelet-rich plasma gel in promoting wound healing based on medical images of wounds. Contrast Media Mol Imaging (2022) 2022:1543604. doi: 10.1155/2022/1543604 36176925 PMC9499777

[181] do Amaral RJFC Zayed NMA Pascu EI Cavanagh B Hobbs C Santarella F. Functionalising collagen-based scaffolds with platelet-rich plasma for enhanced skin wound healing potential. Front Bioeng Biotechnol (2019) 7:371. doi: 10.3389/fbioe.2019.00371 31921799 PMC6915093

[182] Pourmoussa A Gardner DJ Johnson MB Wong AK. An update and review of cell-based wound dressings and their integration into clinical practice. Ann Transl Med (2016) 4(23):457. doi: 10.21037/atm.2016.12.44 28090513 PMC5220025

[183] Syed O Walters NJ Day RM Kim HW Knowles JC. Evaluation of decellularization protocols for production of tubular small intestine submucosa scaffolds for use in oesophageal tissue engineering. Acta Biomater (2014) 10:12. doi: 10.1016/j.actbio.2014.08.024 25173840

[184] Zhao Y Fan J Bai S. Biocompatibility of injectable hydrogel from decellularized human adipose tissue in *vitro* and in vivo. J BioMed Mater Res B Appl Biomater (2019) 107:5. doi: 10.1002/jbm.b.34261 30352138

[185] Greco KV Francis L Somasundaram M Greco G English NR Roether JA. Characterisation of porcine dermis scaffolds decellularised using a novel non-enzymatic method for biomedical applications. J Biomater Appl (2015) 30:2. doi: 10.1177/0885328215578638 25855682

[186] Woodrow T Chant T Chant H. Treatment of diabetic foot wounds with acellular fish skin graft rich in omega-3: a prospective evaluation. J Wound Care (2019) 28:2. doi: 10.12968/jowc.2019.28.2.76 30767649

[187] Hsieh CM Wang W Chen YH Wei PS Liu YH Sheu MT. A novel composite hydrogel composed of formic acid-decellularized pepsin-soluble extracellular matrix hydrogel and sacchachitin hydrogel as wound dressing to synergistically accelerate diabetic wound healing. Pharmaceutics (2020) 12:6. doi: 10.3390/pharmaceutics12060538 PMC7357096 32545186

[188] Tracy LE Minasian RA Caterson EJ. Extracellular matrix and dermal fibroblast function in the healing wound. Adv Wound Care (New Rochelle) (2016) 5:3. doi: 10.1089/wound.2014.0561 PMC4779293 26989578

[189] Seth N Chopra D Lev-Tov H. Fish skin grafts with omega-3 for treatment of chronic wounds: exploring the role of omega-3 fatty acids in wound healing and A review of clinical healing outcomes. Surg Technol Int (2022) 40:38–46. doi: 10.52198/22.STI.40 35483381

[190] Cazzell S Vayser D Pham H Walters J Reyzelman A Samsell B. A randomized clinical trial of a human acellular dermal matrix demonstrated superior healing rates for chronic diabetic foot ulcers over conventional care and an active acellular dermal matrix comparator. Wound Repair Regener (2017) 25:3. doi: 10.1111/wrr.12551 28544150

[191] Wang W PF Li Xie R XJ Ju Liu Z Chu LY. Designable micro-/nano-structured smart polymeric materials. Adv Mater (2022) 34(46):e2107877. doi: 10.1002/adma.202107877 34897843

[192] Elzayat A Adam-Cervera I Álvarez-Bermúdez O Muñoz-Espí R Elzayat A Adam-Cervera I. Nanoemulsions for synthesis of biomedical nanocarriers Nanoemulsions for synthesis of biomedical nanocarriers. Colloids Surf B Biointerfaces Colloids Surf B Biointerfaces (2021) 203:111764. doi: 10.1016/j.colsurfb.2021.111764 33892282

[193] Genesi BP de Melo Barbosa R Severino P Rodas ACD Yoshida CMP Mathor MB. Aloe vera and copaiba oleoresin-loaded chitosan films for wound dressings: microbial permeation, cytotoxicity, and in *vivo* proof of concept. Int J Pharm (2023) 634:122648. doi: 10.1016/j.ijpharm.2023.122648 36709832

[194] Yeo E Yew Chieng CJ Choudhury H Pandey M Gorain B. Tocotrienols-rich naringenin nanoemulgel for the management of diabetic wound: Fabrication, characterization and comparative in *vitro* evaluations. Curr Res Pharmacol Drug Discovery (2021) 2:100019. doi: 10.1016/j.crphar.2021.100019 PMC8663980 34909654

[195] Mariadoss AVA Sivakumar AS Lee CH Kim SJ. Diabetes mellitus and diabetic foot ulcer: Etiology, biochemical and molecular based treatment strategies via gene and nanotherapy. BioMed Pharmacother (2022) 151:113134. doi: 10.1016/j.biopha.2022.113134 35617802

[196] Liu C Zeng H Chen Z Ge Z Wang B Liu B. Sprayable methacrylic anhydride-modified gelatin hydrogel combined with bionic neutrophils nanoparticles for scar-free wound healing of diabetes mellitus. Int J Biol Macromol (2022) 202:418–30. doi: 10.1016/j.ijbiomac.2022.01.083 35051497

[197] Tabriz AG Douroumis D Boateng J. 3D printed scaffolds for wound healing and tissue regeneration. Ther Dress Wound Healing Appl (2020) 2020:385–98. doi: 10.1002/9781119433316.ch17

[198] Baltazar T Merola J Catarino C Xie CB Kirkiles-Smith NC Lee V. Three dimensional bioprinting of a vascularized and perfusable skin graft using human keratinocytes, fibroblasts, pericytes, and endothelial cells. Tissue Eng Part A (2020) 26(5-6):227–38. doi: 10.1089/ten.TEA.2019.0201 PMC7476394 31672103

[199] Zhao M Wang J Zhang J Huang J Luo L Yang Y. Functionalizing multi-component bioink with platelet-rich plasma for customized *in-situ* bilayer bioprinting for wound healing. Mater Today Bio (2022) 16. doi: 10.1016/j.mtbio.2022.100334 PMC9254123 35799896

[200] Albanna M Binder KW Murphy SV Kim J Qasem SA Zhao W. *In situ* bioprinting of autologous skin cells accelerates wound healing of extensive excisional full-thickness wounds. Sci Rep (2019) 9:1. doi: 10.1038/s41598-018-38366-w 30755653 PMC6372693

[201] Jin R Cui Y Chen H Zhang Z Weng T Xia S. Three-dimensional bioprinting of a full-thickness functional skin model using acellular dermal matrix and gelatin methacrylamide bioink. Acta Biomater (2021) 131:248–261. doi: 10.1016/j.actbio.2021.07.012 34265473

[202] Zhou F Hong Y Liang R Zhang X Liao Y Jiang D. Rapid printing of bio-inspired 3D tissue constructs for skin regeneration. Biomaterials (2020) 258. doi: 10.1016/j.biomaterials.2020.120287 32847683

[203] Kim N Lee H Han G Kang M Park S DE K. 3D-printed functional hydrogel by DNA-induced biomineralization for accelerated diabetic wound healing. Adv Sci (Weinh) (2023) 10(17):e2300816. doi: 10.1002/advs.202300816 37076933 PMC10265106

[204] Kędzierska M Bańkosz M Potemski P. Studies on the impact of the photoinitiator amount used during the PVP-based hydrogels’ Synthesis on their physicochemical properties. Materials (Basel) (2022) 15:17. doi: 10.3390/ma15176089 PMC9457623 36079469

[205] Koyuncu A Koç S Akdere ÖE Çakmak AS Gümüşderelioğlu M. Investigation of the synergistic effect of platelet-rich plasma and polychromatic light on human dermal fibroblasts seeded chitosan/gelatin scaffolds for wound healing. J Photochem Photobiol B (2022) 232. doi: 10.1016/j.jphotobiol.2022.112476 35633608

[206] Tong C Zhong X Yang Y Liu X Zhong G Xiao C. PB@PDA@Ag nanosystem for synergistically eradicating MRSA and accelerating diabetic wound healing assisted with laser irradiation. Biomaterials (2020) 243. doi: 10.1016/j.biomaterials.2020.119936 32171103

[207] Huang S Xu S Hu Y Zhao X Chang L Chen Z. Preparation of NIR-responsive, ROS-generating and antibacterial black phosphorus quantum dots for promoting the MRSA-infected wound healing in diabetic rats. Acta Biomater (2022) 137:199–217. doi: 10.1016/j.actbio.2021.10.008 34644613

[208] Qiao Y Ping Y Zhang H Zhou B Liu F Yu Y. Laser-activatable cuS nanodots to treat multidrug-resistant bacteria and release copper ion to accelerate healing of infected chronic nonhealing wounds. ACS Appl Mater Interfaces (2019) 11:4. doi: 10.1021/acsami.8b21766 PMC6727190 30605311

[209] Zhang Y Chen W Feng W Fang W Han X Cheng C. Multifunctional chondroitin sulfate based hydrogels for promoting infected diabetic wounds healing by chemo-photothermal antibacterial and cytokine modulation. Carbohydr Polym. (2023) 314:120937. doi: 10.1016/j.carbpol.2023.120937 37173033

[210] Farber PL Isoldi FC Ferreira LM. Electric factors in wound healing. Adv Wound Care (New Rochelle) (2021) 10(8):461–76. doi: 10.1089/wound.2019.1114 PMC8236302 32870772

[211] Kiaee G Mostafalu P Samandari M Sonkusale S. A pH-mediated electronic wound dressing for controlled drug delivery. Adv Healthc Mater (2018) 7:18. doi: 10.1002/adhm.201800396 30073801

[212] Maijer A Gessner A Trumpatori B Varhus JD. Bioelectric dressing supports complex wound healing in small animal patients. Top Companion Anim Med (2018) 33:1. doi: 10.1053/j.tcam.2018.02.001 29793725

[213] Li P Xu J Shi Q Wang J Zhang W Zheng L. Pulse capacitive coupling electric field regulates cell migration, proliferation, polarization, and vascularization to accelerate wound healing. Adv Wound Care (New Rochelle) (2023) 12(9):498–512. doi: 10.1089/wound.2021.0194 36355602

[214] Bao L Park J Bonfante G Kim B. Recent advances in porous microneedles: materials, fabrication, and transdermal applications. Drug Deliv Transl Res (2022) 12:2. doi: 10.1007/s13346-021-01045-x PMC8724174 34415566

[215] Lim DJ Kim HJ. Microneedles in action: microneedling and microneedles-assisted transdermal delivery. Polymers (Basel) (2022) 14(8):1608. doi: 10.3390/polym14081608 35458358 PMC9024532

[216] Yuan M Liu K Jiang T Li S Chen J Wu Z. GelMA/PEGDA microneedles patch loaded with HUVECs-derived exosomes and Tazarotene promote diabetic wound healing. J Nanobiotechnol (2022) 20(1):147. doi: 10.1186/s12951-022-01354-4 PMC8934449 35305648

[217] Wang Y Lu H Guo M Chu J Gao B He B. Personalized and programmable microneedle dressing for promoting wound healing. Adv Healthc Mater (2022) 11:2. doi: 10.1002/adhm.202101659 34699675

[218] Guo M Wang Y Gao B He B. Shark tooth-inspired microneedle dressing for intelligent wound management. ACS Nano (2021) 15:9. doi: 10.1021/acsnano.1c06279 34533924

[219] Wang Y Gao B He B. Toward efficient wound management: bioinspired microfluidic and microneedle patch. Small (2022) 19:3. doi: 10.1002/smll.202206270 36464498

[220] Zhang J Liu H Yu Q Zhan Z Li T Shu L. Hair derived microneedle patches for both diabetic foot ulcer prevention and healing. ACS Biomater Sci Eng (2023) 9(1):363–74. doi: 10.1021/acsbiomaterials.2c01333 36564012

[221] Guo Z Liu H Shi Z Lin L Li Y Wang M. Responsive hydrogel-based microneedle dressing for diabetic wound healing. J Mater Chem B (2022) 10:18. doi: 10.1039/d2tb00126h 35416225

[222] Ning T Yang F Chen D Jia Z Yuan R Du Z. Synergistically detachable microneedle dressing for programmed treatment of chronic wounds. Adv Healthc Mater (2022) 11:11. doi: 10.1002/adhm.202102180 35133082

[223] Loncar-Turukalo T Zdravevski E MaChado da Silva J Chouvarda I Trajkovik V. Literature on wearable technology for connected health: scoping review of research trends, advances, and barriers. J Med Internet Res (2019) 21:9. doi: 10.2196/14017 PMC6818529 31489843

[224] He X Yang S Liu C Xu T Zhang X. Integrated wound recognition in bandages for intelligent treatment. Adv Healthc Mater (2020) 9:22. doi: 10.1002/adhm.202000941 33015983

[225] Liu Z Liu J Sun T Zeng D Yang C Wang H. Integrated multiplex sensing bandage for *in situ* monitoring of early infected wounds. ACS Sens (2021) 6:8. doi: 10.1021/acssensors.1c01279 34347450

[226] Mariani F Serafini M Gualandi I Arcangeli D Decataldo F Possanzini L. Advanced wound dressing for real-time pH monitoring. ACS Sens (2021) 6:6. doi: 10.1021/acssensors.1c00552 PMC8294608 34076430

[227] Kalasin S Sangnuang P Surareungchai W. Intelligent wearable sensors interconnected with advanced wound dressing bandages for contactless chronic skin monitoring: artificial intelligence for predicting tissue regeneration. Anal Chem (2022) 94:18. doi: 10.1021/acs.analchem.2c00782 35467846

[228] Jiang Y Trotsyuk AA Niu S Henn D Chen K Shih CC. Wireless, closed-loop, smart bandage with integrated sensors and stimulators for advanced wound care and accelerated healing. Nat Biotechnol (2023) 41(5):652–62. doi: 10.1038/s41587-022-01528-3 36424488

[229] Ullah I Wagih M Sun Y Li Y Hajdu K Courson R. Wirelessly powered drug-free and anti-infective smart bandage for chronic wound care. IEEE Trans BioMed Circuits Syst (2023). doi: 10.1109/TBCAS.2023.3277318 37204964

[230] Pappachan JM Cassidy B Fernandez CJ Chandrabalan V Yap MH. The role of artificial intelligence technology in the care of diabetic foot ulcers: the past, the present, and the future. World J Diabetes (2022) 13(12):1131–9. doi: 10.4239/wjd.v13.i12.1131 PMC9791570 36578875

[231] McKinley KL Longaker MT Naik S. Emerging frontiers in regenerative medicine. Science (2023) 380(6647):796–8. doi: 10.1126/science.add6492 PMC10493035 37228215

[232] Kulkarni PG Paudel N Magar S Santilli MF Kashyap S Baranwal AK. Overcoming challenges and innovations in orthopedic prosthesis design: an interdisciplinary perspective. BioMed Mater Devices (2023) 12:1–12. doi: 10.1007/s44174-023-00087-8 PMC10180679 37363137

[233] Adib Y Bensussan A Michel L. Cutaneous wound healing: A review about innate immune response and current therapeutic applications. Mediators Inflammation (2022) 2022. doi: 10.1155/2022/5344085 PMC9061066 35509434

[234] Mascharak S Talbott HE Januszyk M Griffin M Chen K Davitt MF. Multi-omic analysis reveals divergent molecular events in scarring and regenerative wound healing. Cell Stem Cell (2022) 29:2. doi: 10.1016/j.stem.2021.12.011 PMC8988390 35077667

[235] Lin CJ Lan YM Ou MQ Ji LQ Lin SD. Expression of miR-217 and HIF-1α/VEGF pathway in patients with diabetic foot ulcer and its effect on angiogenesis of diabetic foot ulcer rats. J Endocrinol Invest (2019) 42:11. doi: 10.1007/s40618-019-01053-2 31079353

[236] Zhu Y Wang Y Jia Y Xu J Chai Y. Roxadustat promotes angiogenesis through HIF-1α/VEGF/VEGFR2 signaling and accelerates cutaneous wound healing in diabetic rats. Wound Repair Regener (2019) 27:4. doi: 10.1111/wrr.12708 30817065

[237] Zhang H Nie X Shi X Zhao J Chen Y Yao Q. Regulatory mechanisms of the wnt/β-catenin pathway in diabetic cutaneous ulcers. Front Pharmacol (2018) 9:1114. doi: 10.3389/fphar.2018.01114 30386236 PMC6199358

[238] Xie L Zhai R Chen T Gao C Xue R Wang N. Panax notoginseng ameliorates podocyte EMT by targeting the wnt/β-catenin signaling pathway in STZ-induced diabetic rats. Drug Des Devel Ther (2020) 14:527–538. doi: 10.2147/DDDT.S235491 PMC7008200 32103895

[239] Marquard FE Jücker M. PI3K/AKT/mTOR signaling as a molecular target in head and neck cancer. Biochem Pharmacol (2020) 172. doi: 10.1016/j.bcp.2019.113729 31785230

[240] Wang J Zhao X Tian G Liu X Gui C Xu L. Down-Regulation of miR-138 Alleviates Inflammatory Response and Promotes Wound Healing in Diabetic Foot Ulcer Rats via Activating PI3K/AKT Pathway and hTERT. Diabetes Metab Syndr Obes (2022) 15:1153–1163. doi: 10.2147/DMSO.S359759 PMC9015052 35444435

[241] Wei F Wang A Wang Q Han W Rong R Wang L. Plasma endothelial cells-derived extracellular vesicles promote wound healing in diabetes through YAP and the PI3K/Akt/mTOR pathway. Aging (Albany NY) (2020) 12:12. doi: 10.18632/aging.103366 PMC7343472 32570219

[242] Hu HH Chen DQ Wang YN Feng YL Cao G Vaziri ND. New insights into TGF-β/Smad signaling in tissue fibrosis. Chem Biol Interact (2018) 292. doi: 10.1016/j.cbi.2018.07.008 30017632

[243] Geng K Ma X Jiang Z Gu J Huang W Wang W. WDR74 facilitates TGF-β/Smad pathway activation to promote M2 macrophage polarization and diabetic foot ulcer wound healing in mice. Cell Biol Toxicol (2022). doi: 10.1007/s10565-022-09748-8 35982296

[244] Qiao Y Yan W He J Liu X Zhang Q Wang X. Identification, evolution and expression analyses of mapk gene family in Japanese flounder (Paralichthys olivaceus) provide insight into its divergent functions on biotic and abiotic stresses response. Aquat Toxicol (2021) 241. doi: 10.1016/j.aquatox.2021.106005 34731643

[245] Zhu W Fang Q Liu Z Chen Q. Novel genes potentially involved in fibroblasts of diabetic wound. J Diabetes Res (2021) 2021. doi: 10.1155/2021/7619610 PMC8670931 34917686

[246] Lawrence T. The nuclear factor NF-kappaB pathway in inflammation. Cold Spring Harb Perspect Biol (2009) 1:6. doi: 10.1101/cshperspect PMC2882124 20457564

[247] Sun X Wang X Zhao Z Chen J Li C Zhao G. Paeoniflorin inhibited nod-like receptor protein-3 inflammasome and NF-κB-mediated inflammatory reactions in diabetic foot ulcer by inhibiting the chemokine receptor CXCR2. Drug Dev Res (2021) 82:3. doi: 10.1002/ddr.21763 33236457

[248] Deng L Du C Song P Chen T Rui S Armstrong DG. The role of oxidative stress and antioxidants in diabetic wound healing. Oxid Med Cell Longev (2021) 2021. doi: 10.1155/2021/8852759 PMC7884160 33628388

[249] Sun X Wang X Zhao Z Chen J Li C Zhao G. Paeoniflorin accelerates foot wound healing in diabetic rats though activating the Nrf2 pathway. Acta Histochem (2020) 122:8. doi: 10.1016/j.acthis.2020.151649 33166863

